# A Phenanthroline-Based Fluorescent Probe for Highly Selective Detection of Extreme Alkalinity (pH > 14) in Aqueous Solution

**DOI:** 10.1186/s11671-019-3149-x

**Published:** 2019-09-18

**Authors:** Xiaoyu Ma, Shanyong Chen, Hong Yu, Youwei Guan, Junjun Li, Xingwu Yan, Zhenghao Zhang

**Affiliations:** 1 Research Institute for New Materials Technology, Chongqing University of Arts and Sciences, Yongchuan, 402160 People’s Republic of China; 2 College of Chemistry, Jilin University, Changchun, 130012 People’s Republic of China; 3 State Grid Tianjin Electric Power Corporation Chengxi District Supply Company, Tianjin, 300191 People’s Republic of China

**Keywords:** Fluorescent probe, Phenanthroline, Extreme alkalinity, Water-soluble, Aggregation-induced enhanced emission

## Abstract

Although numerous fluorescent probes are designed to detect the pH value in the past decades, developing fluorescent probes for extreme alkalinity (pH > 14) detection in aqueous solution is still a great challenge. In this work, we utilized 1H-imidazo[4,5-f][1, 10] phenanthroline (IP) group as the recognition group of hydroxyl ion and introduced two triethylene glycol monomethyl ether groups to improve its solubility. This IP derivative, BMIP, possessed good solubility (25 mg/mL) in water. It displayed high selectivity toward extreme alkalinity (pH > 14) over other ions and pH (from extreme acidity to pH = 14). From 3 to 6 mol/L OHˉ, the exact concentration of OHˉ could be revealed by BMIP and the whole detection process just needed a short time (≤ 10 s). Meanwhile, it exhibited good anti-interference ability and repeatability during the detection process. Through optical spectra and NMR analysis, its detection mechanism was proved to be deprotonation by hydroxyl ion and then aggregation-induced enhanced emission. Our study presents a new basic group based on which researchers can develop new fluorescent probes that can detect extreme alkalinity (pH > 14) in aqueous solution.

## Introduction

For a paper-making industry, nuclear fuel reprocessing, waste and waste water treatment, leatherworking, metal mining, and microbial production process, extreme alkaline (pH > 14) condition is necessary [1–3]. To ensure the pH value at extreme alkaline region, monitoring the pH value of these processes is essential. In the past decades, researchers have developed many methods to detect the pH value, such as pH test paper and pH electrode [4–9]. However, common detection methods are not suitable for extreme alkalinity (pH > 14). At extreme alkaline region (pH > 14), the pH test paper shows a dark blue color irrespective of the hydroxide concentration and the pH electrode cannot give correct values. To solve this problem, researchers introduced fluorescent probes and this method had been proved to be feasible [10]. But overall, most of fluorescent probes were designed to detect weak acidity or alkalinity whose pH values were between 2 and 13, while little attention was paid to fluorescent probes in low (pH < 2) or high pH (pH > 13) regions [11–23]. For this reason, the performance of present fluorescent probes cannot meet the requirement of the above production processes. Therefore, developing fluorescent probes which can detect extreme alkalinity (pH > 14) effectively is eager.

In extreme alkalinity detection filed, Thakur [10], Khalil [24], Xue [25–27], and Sadik [28] carried out pioneering and excellent work. At present, several fluorescent probes which can detect extreme alkalinity (pH > 14) have been reported [8, 22–26]. However, studies in this filed are still in the initial stage and many problems exist, such as (1) fluorescent probes which can detect pH > 14 are rare, (2) most of these fluorescent probes need organic solvents to assist their detections and few fluorescent probes can detect extreme alkalinity in pure water [22, 24, 25], and (3) for many fluorescent probes, the principle of sensing extreme alkalinity is measuring their absorbance changes and this brings about low sensitivity [22, 23, 26]. To improve the above situation, designing fluorescent probes with high sensitivity and the ability to detect pH > 14 in aqueous solution is necessary.

1H-imidazo[4,5-f] [1, 10] phenanthroline (IP), a rigid planar group, possesses high charge transporting ability and good fluorescent properties. Therefore, its derivatives were widely used in organic light-emitting diodes, organic thin-film transistors, and many other fields [29, 30]. Compared to these applications, its application for extreme alkalinity (pH > 14) detection has never been reported. However, this group has the potential of acting as a good probe for detecting extreme alkalinity (pH > 14) because of the following reasons: (1) it has NH group which can react with hydroxyl ion, and therefore, it can be used as the recognition group of hydroxyl ion; (2) its good fluorescent property can endow the probe with high sensitivity; (3) compared with common organic aromatic groups which almost have no solubility in water, IP group has weak solubility in water which is favorable for designing water-soluble fluorescent probes further. Because of these advantages, from IP group, it was possible to develop new water-soluble fluorescent probes with high sensitivity for extreme alkalinity detection. These new probes can solve the above problems which exist in previous probes. This is eager for this field.

**Fig. 1:**
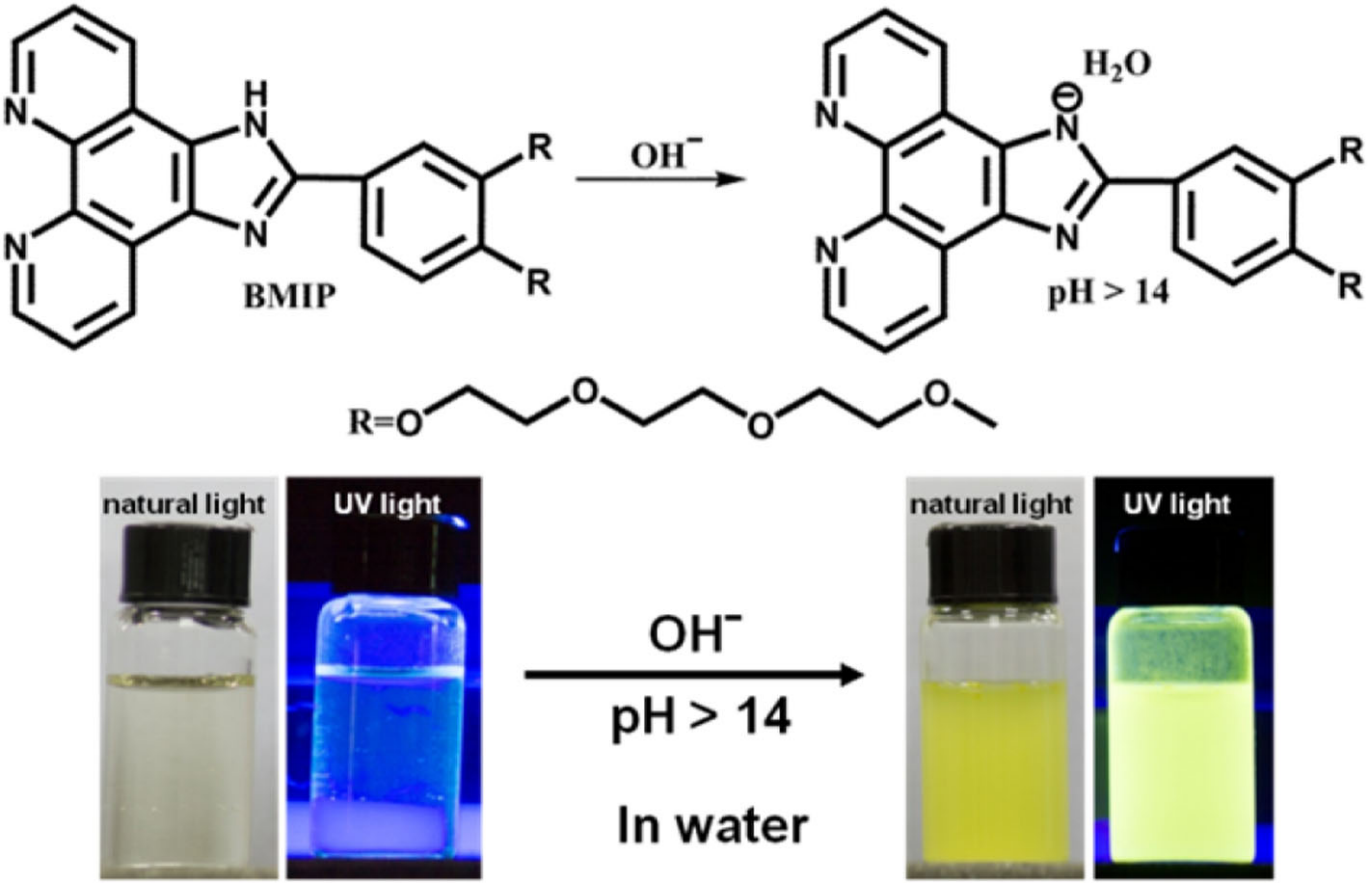
The detection mechanism for extreme alkalinity (pH > 14); photos shot under natural light (left) and UV light (365 nm) (right)

Hence, in this work, we utilized the IP group to design fluorescent probe for the detection of extreme alkalinity (pH > 14). We introduced two triethylene glycol monomethyl ether groups to improve the solubility of this probe and obtained an IP derivative, BMIP (Fig. 1). The preparation and solubility of BMIP were studied. Its selectivities and detectabilities for extreme alkalinity (pH > 14) were carefully examined. In addition, we also studied its detection mechanism through optical spectra and NMR spectrum.

## Results and Discussion

### Syntheses, Solubility, and Detection Concentration of BMIP

**Scheme 1:**
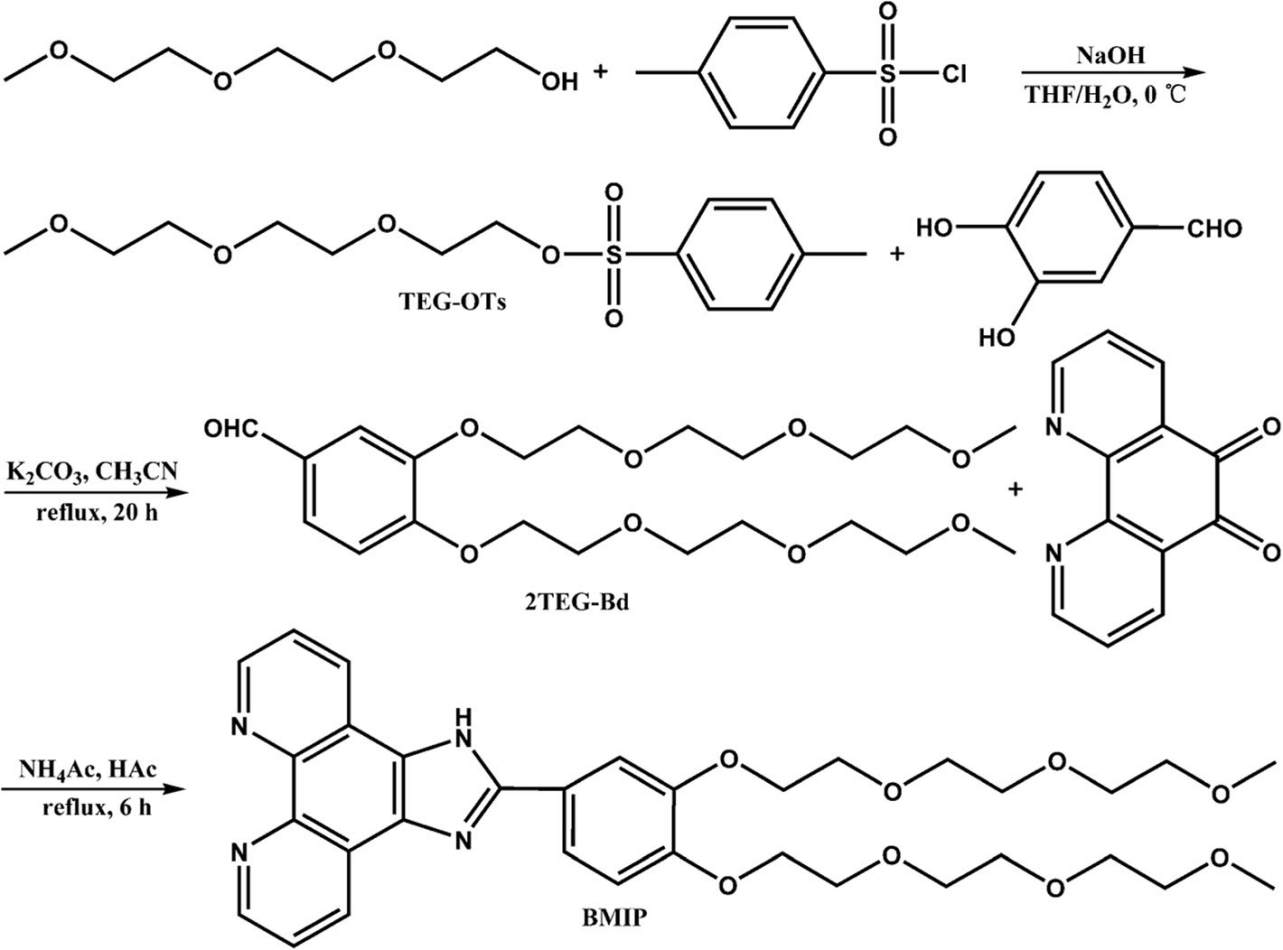
Synthetic procedures and structures of compounds

After three steps, BMIP was obtained from triethylene glycol monomethyl ether and 1,10-phenanthroline-5,6-dione (Scheme 1). The crude product was further purified by extraction and column chromatography to obtain a light-red gelatinous sample. BMIP exhibited excellent solubility in organic solvents and water. In water, its solubility was as high as 25 mg/mL which meant it could work well in pure water.

To determine the most suitable concentration for detections, we prepared aqueous solutions of BMIP with different concentrations (2 × 10^−5^, 2 × 10^−4^, 2 × 10^−3^, 4 × 10^−3^ mol/L) first. Then, sodium hydroxide (NaOH) solution (6 mol/L, 2 mL) was added to these solutions (2 mL), respectively. After that, the absorption and fluorescence spectra of these mixtures were studied. Results were shown in Additional file 1: Figures S1, S2, and S3. For BMIP, at the concentration of 10^−5^ mol/L, its response to extreme alkalinity was weak (Additional file 1: Figure S1). At the concentration of 10^−4^ and 2 × 10^−3^ mol/L, its response to extreme alkalinity was good but its response sensitivity for different alkalinities could not meet the requirement of detections (Additional file 1: Figure S1). Finally, 10^−3^ mol/L (1 mmol/L) was determined to be the best concentration of BMIP for detection because the response sensitivity was good at this concentration.

But at this concentration (1 mmol/L), the absorption intensities of those solutions below exceeded the measuring range of equipment (we tried four absorption spectrophotometers and the results were the same). Because of the limit of measuring equipment, it was regretful that the changes of absorption spectra during those experiments below were not clear (Additional file 1: Figure S2, S5, S8, and S14).

### Ion Selectivities and Anti-Interference Ability

**Fig. 2:**
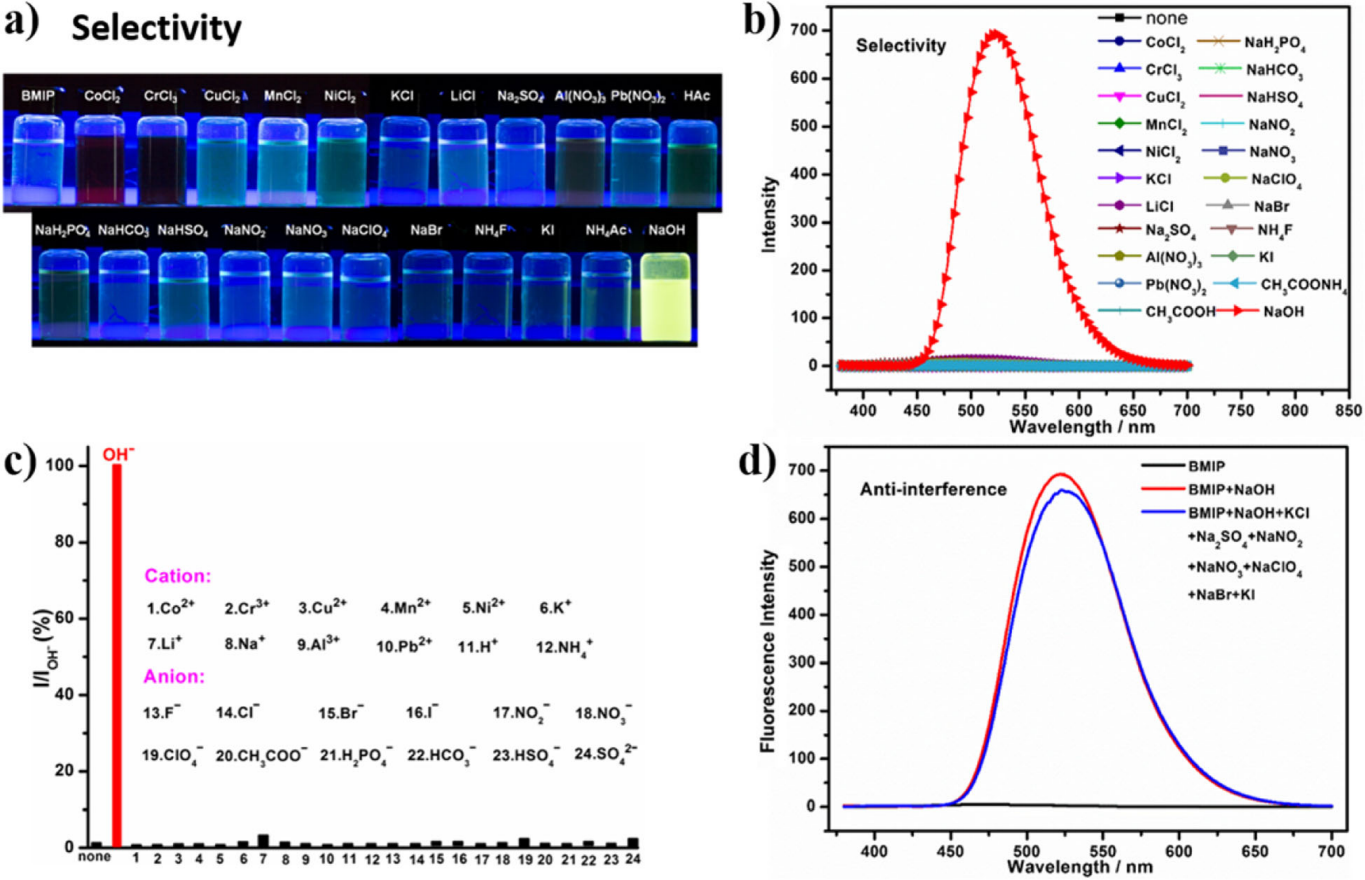
**a** Photo comparison (under UV light) (365 nm) and **b** the PL spectra of aqueous solutions (1 mmol/L) of BMIP before and after the additions of different salts (CoCl_2_, CrCl_3_, CuCl_2_, MnCl_2_, NiCl_2_, KCl, LiCl, Na_2_SO_4_, Al (NO_3_)_3_, Pb (NO_3_)_2_, CH_3_COOH, NaH_2_PO_4_, NaHCO_3_, NaHSO_4_, NaNO_2_, NaNO_3_, NaClO_4_, NaBr, NH_4_F, KI, CH_3_COONH_4_, NaOH, respectively) (3 mol/L). **c** The I/I_OHˉ_ ratios of fluorescence responses of BMIP solutions (1 mmol/L) before and after the additions of different ions (Co^2+^, Cr^3+^, Cu^2+^, Mn^2+^, Ni^2+^, K^+^, Li^+^, Na^+^, Al^3+^, Pb^2+^, H^+^, NH_4_^+^, Fˉ, Clˉ, Brˉ, Iˉ, NO_2_^ˉ^, NO_3_ˉ, ClO_4_ˉ, CH_3_COOˉ, H_2_PO_4_ˉ, HCO_3_ˉ, HSO_4_ˉ, SO_4_^2^ˉ, and OHˉ, respectively) (3 mol/L) in water (*I*_OHˉ_ represents the fluorescence intensity of BMIP solution after the addition of OHˉ (3 mol/L), *I* represents the fluorescence intensities of BMIP solution before and after the additions of other ions). **d** The PL spectra of aqueous solutions (1 mmol/L) of BMIP at different conditions (black line, BMIP solution without any additives; red line, BMIP solution after adding NaOH (3 mol/L); blue line, BMIP solution after adding NaOH, KCl, Na_2_SO_4_, NaNO_2_, NaNO_3_, NaClO_4_, NaBr, and KI (3 mol/L))

For a good fluorescent probe, it should have high selectivity toward specific ions over other competitive ions. To investigate the selectivity of BMIP, we added different salts (CoCl_2_, CrCl_3_, CuCl_2_, MnCl_2_, NiCl_2_, KCl, LiCl, Na_2_SO_4_, Al (NO_3_)_3_, Pb (NO_3_)_2_, CH_3_COOH, NaH_2_PO_4_, NaHCO_3_, NaHSO_4_, NaNO_2_, NaNO_3_, NaClO_4_, NaBr, NH_4_F, KI, CH_3_COONH_4_, NaOH, respectively) to the aqueous solutions of BMIP and then studied the changes of its color and fluorescence (Fig. 2 and Additional file 1: Figure S4).

When NaOH was added and the pH value of BMIP solution was higher than 14 (pH > 14), the BMIP solution changed immediately from almost colorless to orange-yellow (Additional file 1: Figure S4), and its fluorescence altered from non-luminous to intensively yellow (525 nm) (Fig. 2a). By contrast, other competitive ions (Co^2+^, Cr^3+^, Cu^2+^, Mn^2+^, Ni^2+^, K^+^, Li^+^, Na^+^, Al^3+^, Pb^2+^, H^+^, NH_4_^+^, Fˉ, Clˉ, Brˉ, Iˉ, NO_2_^ˉ^, NO_3_ˉ, ClO_4_ˉ, CH_3_COOˉ, H_2_PO_4_ˉ, HCO_3_ˉ, HSO_4_ˉ, and SO_4_^2^ˉ) almost did not bring about obvious fluorescence changes for BMIP solution (Fig. 2b, c). Compared to extreme alkalinity (pH > 14), the slight changes of fluorescence intensities caused by some competitive ions could be ignored (Fig. 2c). Therefore, BMIP exhibited high selectivity toward extreme alkalinity (pH > 14) over other ions.

To investigate the anti-interference ability of BMIP, several salts (KCl, Na_2_SO_4_, NaNO_2_, NaNO_3_, NaClO_4_, NaBr, and KI) were added to the mixed solution of BMIP and NaOH. Then, the changes of its fluorescence were studied (Fig. 2d). After the addition of these salts, the fluorescence of the mixed solution almost had no changes except for a slight decrease of fluorescence intensity (Fig. 2d). This indicated that BMIP had good anti-interference ability during the detection process of extreme alkalinity.

### Response to Different pH

**Fig. 3:**
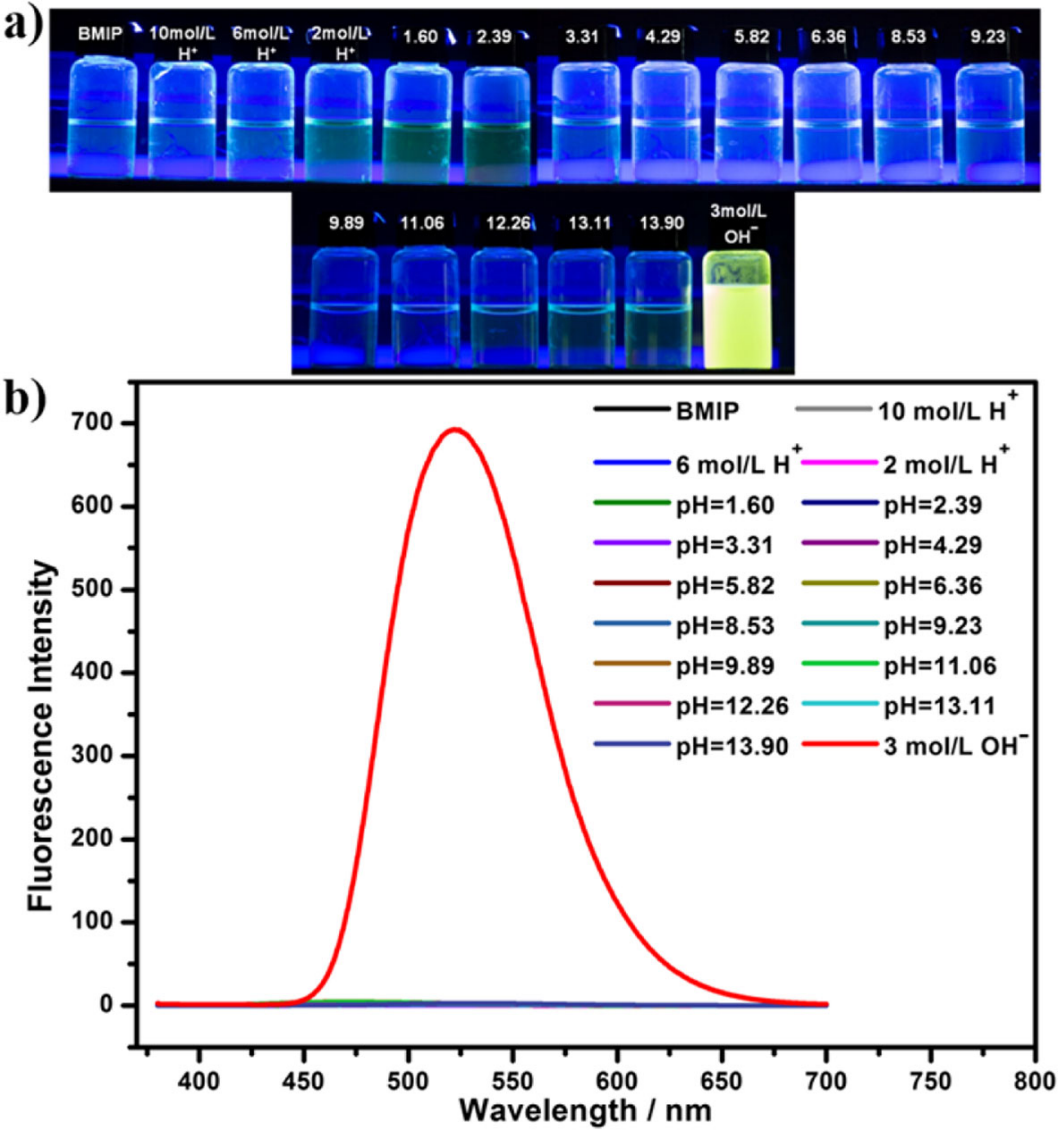
**a** Photo comparison (under UV light) (365 nm) and **b** the PL spectra of aqueous solutions (1 mmol/L) of BMIP with different pH (neutral water, 10 mol/L H^+^, 6 mol/L H^+^, 2 mol/L H^+^, 1.60, 2.39, 3.31, 4.29, 5.82, 6.36, 8.53, 9.23, 9.89, 11.06, 12.26, 13.11, 13.90, 3 mol/L OHˉ, respectively)

The above experiments demonstrated that BMIP had high selectivity toward special pH range (pH > 14). To examine whether BMIP had obvious response to other pH values, we prepared aqueous solutions of BMIP with different pH values (10 mol/L H^+^, 6 mol/L H^+^, 2 mol/L H^+^, 1.60, 2.39, 3.31, 4.29, 5.82, 6.36, 8.53, 9.23, 9.89, 11.06, 12.26, 13.11, 13.90, 3 mol/L OHˉ, respectively) and then studied the color and fluorescence of these solutions (Fig. 3 and Additional file 1: Figure S7).

When the pH value of BMIP solution was below 14 (from 10 mol/L H^+^ to 13.90), the fluorescence had no change and the solutions exhibited non-luminous (Fig. 3). When the pH value of BMIP solution increased to extreme alkalinity (3 mol/L OHˉ), the solution exhibited intensively yellow fluorescence and the fluorescence intensity was almost 1000 times higher than those of other solutions (pH < 14) (Fig. 3). Therefore, for different pH values, BMIP only exhibited a strong response to extreme alkalinity (pH > 14) and had no fluorescent response to other pH values.

### Extreme Alkalinity Detection and Repeatability

Good fluorescent probes should be able to reveal the exact concentration of detected objects. This means there is a mathematical curve relationship between the fluorescence intensity and the concentration of detected objects. To obtain such a mathematical curve, we prepared aqueous solutions of BMIP with different concentrations of OHˉ (0, 1.0, 1.5, 2.0, 2.5, 3.0, 3.5, 4.0, 4.5, 5.0, 5.5, 6.0, 6.5, 7.0, 7.5, 8.0, 8.5, 9.0, 9.5, 10, 10.5, 11, 11.5, 12, 12.5, 13, 13.5, 14, 14.5, 15 mol/L, respectively) and then studied the color and fluorescence of these solutions (Additional file 1: Figures S10, S11, S12, and S13).

**Fig. 4:**
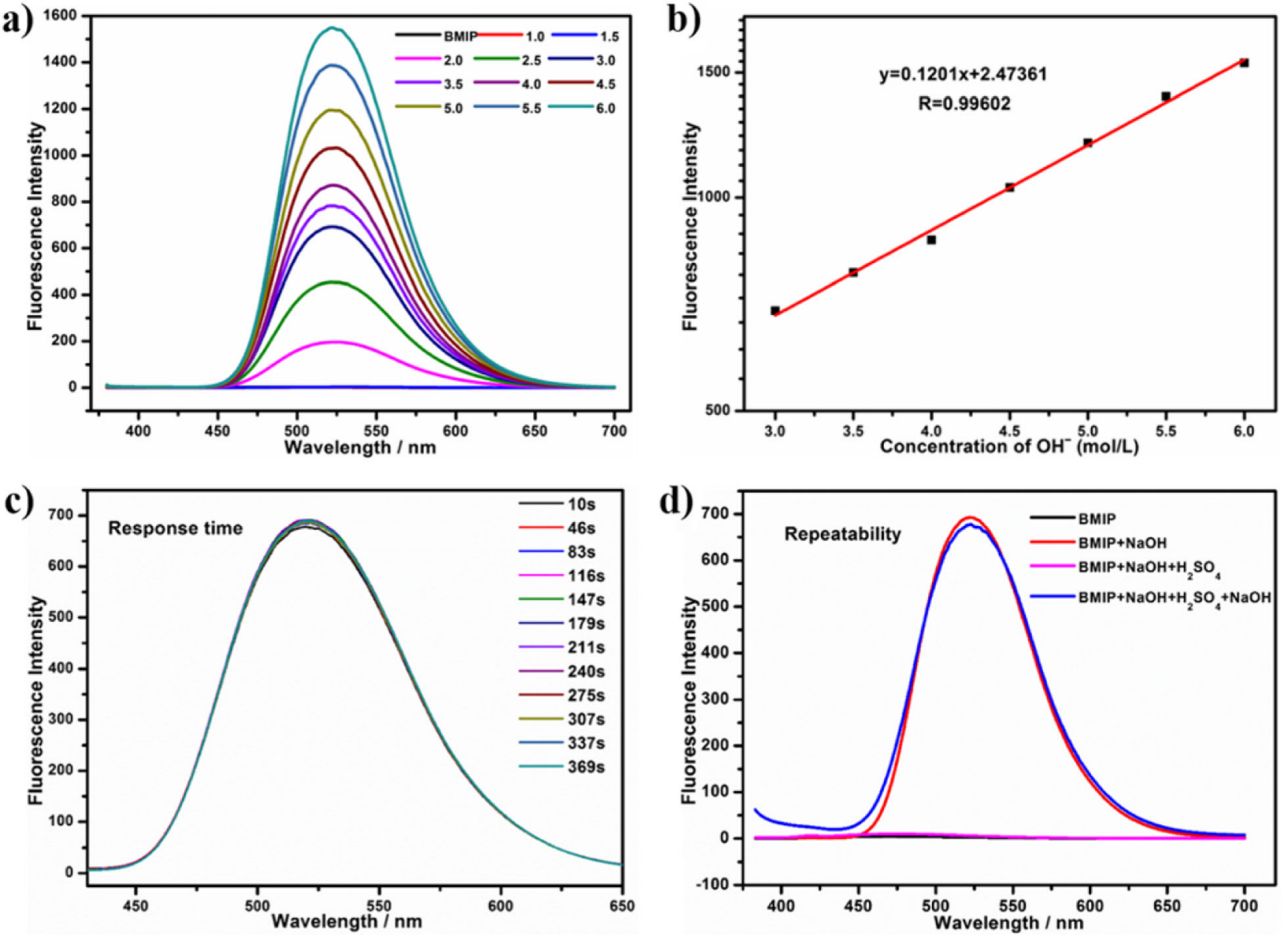
**a** The PL spectra of aqueous solutions (1 mmol/L) of BMIP with different concentrations (0, 1.0, 1.5, 2.0, 2.5, 3.0, 3.5, 4.0, 4.5, 5.0, 5.5, 6.0 mol/L, respectively) of OHˉ. **b** The changes of fluorescence intensities of BMIP solutions (1 mmol/L) with different concentrations (3.0, 3.5, 4.0, 4.5, 5.0, 5.5, 6.0 mol/L, respectively) of OHˉ in water. **c** The PL spectra of aqueous solution (3 mol/L OHˉ) of BMIP (1 mmol/L) at different times (10 s, 46 s, 83 s, 116 s, 147 s, 179 s, 211 s, 240 s, 275 s, 307 s, 337 s, 369 s, respectively). **d** The PL spectra of aqueous solutions (1 mmol/L) of BMIP (3 mL) at different conditions (black line, BMIP solution without any additives; red line, BMIP solution after adding NaOH (3 mol/L); purple line, BMIP solution after adding NaOH (3 mol/L) and then the solution pH became neutral by adding sulfuric acid; blue line, BMIP solution after adding NaOH (3 mol/L), then the solution pH became neutral by adding sulfuric acid and finally adding NaOH (3 mol/L) again)

From 1 to 1.5 mol/L, the color of BMIP solutions had a slight change but their fluorescence almost did not alter (Fig. 4a, Additional file 1: Figures S10, S11, and S12). At the concentration of 2 mol/L, the color and fluorescence of BMIP solution showed a sudden big change. At this concentration, yellow precipitate appeared and the color of BMIP solution altered from almost colorless to orange-yellow (Additional file 1: Figure S10). Meanwhile, the fluorescence changed from non-luminous to intensively yellow (525 nm) and the fluorescence intensity was almost 200 times higher than that of BMIP solution (pH = 7) (Fig. 4a and Additional file 1: Figure S11). From 2 to 6 mol/L, the yellow precipitate gradually increased and the fluorescence was gradually enhanced (Fig. 4a and Additional file 1: Figure S13). From 6 to 8.5 mol/L, the precipitate did not increase and the fluorescence intensity retained a stable level (Additional file 1: Figures S12 and S13). From 9 to 15 mol/L, the amount of precipitate did not change but the precipitate was uniformly dispersed in the solution. This lowered the fluorescence intensity (Additional file 1: Figures S12 and S13).

The whole mathematical curve about the relationship between the fluorescence intensity and the concentration of OHˉ was shown in Additional file 1: Figure S13. In this curve, we discovered that from 3 to 6 mol/L, the plot of fluorescence intensity *vs* the concentration of OHˉ showed good linearity (*R* = 0.99602) (Fig. 4b). Different concentrations of OHˉ were corresponding to different fluorescence intensities. This meant BMIP could reveal the concentration of OHˉ in this range (3–6 mol/L) through measuring the fluorescence intensity (Fig. 4a, b).

To test the detection time of BMIP toward OHˉ, we measured the PL spectra of aqueous solution (3 mol/L OHˉ) of BMIP (1 mmol/L) at different times (10 s, 46 s, 83 s, 116 s, 147 s, 179 s, 211 s, 240 s, 275 s, 307 s, 337 s, 369 s, respectively). From 10 to 369 s, the PL spectra were almost the same except for a slight change of fluorescence intensity (Fig. 4c). This result revealed that BMIP could detect OHˉ (3–6 mol/L) in a short time (≤ 10 s).

To investigate the detection repeatablity of BMIP toward OHˉ, the fluorescence of four different BMIP solution (1 mmol/L) were studied (Fig. 4d). These four solutions (final volume: 3 mL) were as follows: (a) BMIP solution (b) BMIP solution after adding NaOH (3 mol/L), (c) BMIP solution after adding NaOH (3 mol/L) and then the solution pH became neutral by adding sulfuric acid, and (d) BMIP solution after adding NaOH (3 mol/L), then the solution pH became neutral by adding sulfuric acid and finally adding NaOH (3 mol/L) again. When OHˉ was added, the fluorescence of BMIP solution was dramatically enhanced (Fig. 4d). After OHˉ reacted with sulfuric acid and the solution pH became neutral, the solution exhibited non-luminous again (Fig. 4d). Finally, when OHˉ was added again, the same yellow fluorescence appeared subsequently (Fig. 4d). These results indicated that BMIP possessed good repeatablity for detecting extreme alkalinity.

Table 1 compares previous publications and this work about the detection of extreme alkalinity (pH > 14). It can be seen that compared with previous probes, BMIP possesses series of obvious improvements: good water solubility which makes it work well in pure water without any assistance of organic solvents, high sensitivity because of its fluorescent response method, fast response time (≤ 10 s), high selectivity, good anti-interference ability and repeatability, and quantitative detection ability. As we know, the performance of BMIP is best during those probes for extreme alkalinity (pH > 14) detection.

**Table 1:** The comparison between previous works and our work about the detection of extreme *alkalinity (pH* > 14)

Compound	Water solubility	Response method	Response time	Selectivity	Quantitative	Anti-interference ability	Repeatability	Mechanism	Reference
BNTP	No	Absorbance	5 min	Moderate	Capable	Moderate	Capable	Deprotonation	10
T^F^PLPt	No	Absorbance	30 min	/	Incapable	/	/	Ring-opening reaction	24
SiO_2_/ZrO_2_-Nafion composite	No	Absorbance	15 s	/	Capable	/	/	Deprotonation	25–27
PAN-PS	No	Absorbance	< 30 s	/	Capable	/	Capable	Deprotonation	28
BMIP	excellent	Fluorescence	≤ 10 s	High	Capable	Good	Capable	Deprotonation AIE	This work

### Detection Mechanism

**Fig. 5:**
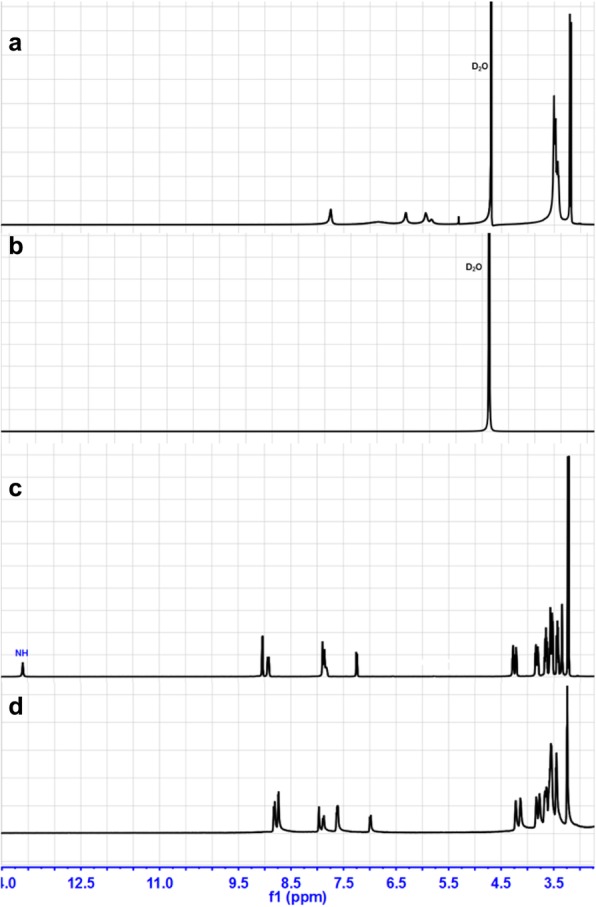
The ^1^H NMR spectrum of BMIP in **a** D_2_O and **c** DMSO-*d*_6_; the ^1^H NMR spectrum of BMIP after the additions of NaOH in **b** D_2_O and **d** DMSO-*d*_6_

Fluorescence transformation between extreme alkaline and natural condition in repeatability experiments indicated that when OHˉ was added, deprotonation might happen and when OHˉ was treated by H^+^, BMIP could recover. To investigate whether deprotonation happened, we measured the ^1^H NMR spectrum of BMIP before and after the addition of NaOH (excessive) (Fig. 5). In D_2_O, after the addition of NaOH, the signals of BMIP disappeared, which revealed the generation of new product (Fig. 5a, b). Then, D_2_O was replaced by DMSO-*d*_6_ to dissolve the precipitate that existed in D_2_O. Obviously, the signal of NH in BMIP disappeared and other signals almost had no changes except for a slight shift of peak position (Fig. 5c, d). Results of repeatability experiments and NMR spectrum revealed that after the addition of OHˉ, deprotonation happened and deprotonated product, BMIPˉ, generated (Fig. 1).

From absorption spectra of BMIP solution at different pH (from 10 mol/L H^+^ to 15 mol/L OHˉ), it could be seen that when the solution pH was higher than 12.26 (pH ≥ 12.26), deprotonation had happened and a new absorption band around 385 nm appeared (Additional file 1: Figures S3, S8, and S14). This meant BMIPˉ had generated at pH ≥ 12.26. However, from 12.26 to 1.5 mol/L OHˉ, BMIPˉ dissolved in water and no obvious fluorescence was observed. At the concentration of 2 mol/L, BMIPˉ precipitated (yellow precipitate) and intensively yellow fluorescence appeared. From 2 to 6 mol/L, with the increase of the concentration of NaOH, the solubility of BMIPˉ in the solution decreased and BMIPˉ gradually precipitated from the aqueous solution (Additional file 1: Figure S10). With the increase of BMIPˉ precipitate, the aggregation of BMIPˉ was gradually enhanced and the fluorescence intensity gradually increased (Fig. 4a, b, Additional file 1: Figures S11, S12, and S13). This was a typical phenomenon of aggregation-induced enhanced emission (AIE). After all of BMIPˉ precipitated from the aqueous solution, the fluorescence intensity would retain a stable level (from 6 to 8.5 mol/L OHˉ) (Additional file 1: Figures S12 and S13). However, when the concentration of NaOH was too high, the high viscosity of aqueous solution would prevent the aggregation of BMIPˉ and then lower the fluorescence intensity (9–15 mol/L OHˉ) (Additional file 1: Figures S12 and S13). These results demonstrated that the variation of fluorescence intensity came from the variation of aggregation degree of BMIPˉ and aggregation-induced enhanced emission was one of the detection mechanisms of BMIP toward extreme alkalinity (pH > 14).

**Fig. 6:**
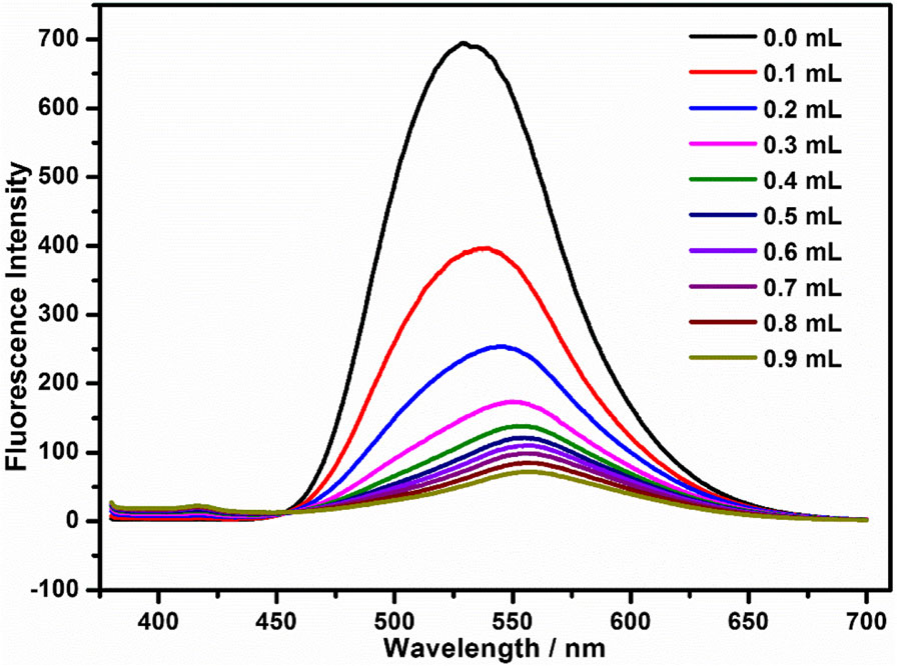
The PL spectra of aqueous solution (3 mol/L OHˉ) (2 mL) of BMIP (1 mmol/L) after the addition of different volumes (0, 0.1, 0.2, 0.3, 0.4, 0.5, 0.6, 0.7, 0.8, and 0.9 mL, respectively) of NaOH solution (3 mol/L)

To further verify the AIE mechanism, BMIP solution (1 mmol/L, 2 mL) with NaOH (3 mol/L) was prepared first, and then, NaOH solution (3 mol/L) was gradually added (0.1 mL every time). During this process, the fluorescence changes of this solution were studied (Fig. 6). With the increase of NaOH solution, some yellow precipitate dissolved and the other precipitate dispersed. Meanwhile, the fluorescence intensity gradually decreased (Fig. 6). This result demonstrated that AIE was one of the detection mechanisms again.

Through the above experiments, the detection mechanism was proved to be deprotonation by hydroxyl ion and then aggregation-induced enhanced emission.

## Conclusion

In summary, our study presented a new recognition group for extreme alkalinity (pH > 14) and a universal group which could greatly improve the water solubility of organic probes. Based on these two groups, a phenanthroline derivative, BMIP, was designed and synthesized. It showed good solubility (25 mg/mL) in water which made it have the ability to work in pure water. In 25 kinds of ions, it exhibited high selectivity toward extreme alkalinity (pH > 14) over other ions. From extreme acidity to extreme alkalinity, it only exhibited a strong response to extreme alkalinity (pH > 14) and had no fluorescent response to other pH values. Meanwhile, during the detection process, it displayed good anti-interference ability and repeatability. From 3 to 6 mol/L OHˉ, the plot of fluorescence intensity *vs* the concentration of OHˉ showed good linearity (*R* = 0.99602) and the concentration of OHˉ could be revealed through measuring the fluorescence intensity. This detection process just needed a short time (≤ 10 s). Finally, its detection mechanism was proved to be deprotonation by hydroxyl ion and then aggregation-induced enhanced emission.

## Methods/Experimental

### General Information

^1^H and ^13^C NMR spectra were measured on a Bruker Avance 400 spectrometer with tetramethylsilane as the internal standard. LC-MS data were recorded with a Shimadzu LCMS-2020. The emission spectra were recorded by a Shimadzu RF-5301 PC spectrometer. All the reagents were commercially available and were directly used as received unless otherwise stated. All reactions were carried out using Schlenk techniques under a nitrogen atmosphere. All of the detection processes were carried out under ambient conditions in pure water.

### Synthesis of BMIP

Synthesis of 2-(2-(2-methoxyethoxy)ethoxy) ethyl 4-methylbenzenesulfonate (TEG-OTs): To a three-neck round-bottom flask, triethyleneglycol monomethyl ether (8 g, 48.6 mmol) and tetrahydrofuran (THF) (15 mL) were added. Then, a solution of NaOH (1.61 g, 0.0414 mol) dissolved in water (15 mL) was added under vigorous stirring. After the mixture was cooled to 0 °C, a solution of tosyl chloride (5.57 g, 0.0292 mol) in THF (15 mL) was dropped slowly. Then, the temperature was raised to room temperature. After 2 h, the mixture was extracted with dichloromethane and the organic layers were washed with an aqueous solution of NaOH (1 M). The organic solvent was removed by rotary evaporation, and the crude product was purified by column chromatography which used dichloromethane first and then dichloromethane/methanol (20:1 v/v) as the eluents. The pure product was a colorless liquid. Yield: 86%. ^1^H NMR (400 MHz, DMSO-*d*_6_, TMS, δ): 2.42 (s, 3H), 3.23 (s, 3H), 3.40–3.49 (m, 8H), 3.57 (t, 2H), 4.11 (t, 2H), 7.48 (d, 2H), 7.79 (d, 2H). ^13^C NMR (400 MHz, DMSO-*d*_6_, TMS, δ): 21.55, 39.42, 39.63, 39.84, 40.05, 40.46, 40.67, 42.23, 58.51, 68.37, 70.06, 70.11, 70.21, 70.44, 71.72, 125.97, 127.19, 128.07, 128.45, 130.25, 130.59, 132.96, 145.34. LC-MS: 319 [M+H]^+^ (calcd: 318.11).

Synthesis of 3,4-bis(2-(2-(2-methoxyethoxy)ethoxy)ethoxy) benzaldehyde (2TEG-Bd): To a two-neck round-bottom flask, 3,4-dihydroxybenzaldehyde (276 mg, 2 mmol), TEG-OTs (1590 mg, 5 mmol), dry potassium carbonate (1382 mg, 10 mmol), and dry acetonitrile (80 mL) were added. Then, the mixture was refluxed under nitrogen atmosphere for 20 h. After the mixture was cooled to room temperature, acetonitrile was removed by rotary evaporation and the solid was dissolved in water. The solution was extracted with dichloromethane for three times (50 mL × 3), and dichloromethane was removed by rotary evaporation successively. The crude product was purified by column chromatography which used ethyl acetate first and then ethyl acetate/methanol (20:1 v/v) as the eluents. The pure product was a light-yellow liquid. Yield: 91%. ^1^H NMR (400 MHz, DMSO-*d*_6_, TMS, δ): 3.23 (s, 6H), 3.41–3.43 (m, 4H), 3.50–3.54 (m, 8H), 3.60–3.63 (m, 4H), 3.78 (dd, 4H), 4.17 (t, 2H), 4.22 (t, 2H), 7.20 (d, 1H), 7.44 (d, 1H), 7.54 (dd, 1H), 9.83 (s, 1H). ^13^C NMR (400 MHz, DMSO-*d*_6_, δ): 38.35, 38.56, 38.77, 38.99, 39.19, 39.40, 39.60, 57.44, 67.78, 67.82, 68.13, 68.26, 69.00, 69.25, 69.45, 69.47, 70.69, 111.49, 112.32, 125.26, 129.20, 147.92, 153.20, 190.72. LC-MS: 431 [M + H]^+^ (calcd: 430.22).

Synthesis of 2-(3,4-bis(2-(2-(2-methoxyethoxy)ethoxy)ethoxy)phenyl)-1H-imidazo[4,5-f] [1, 10] phenanthroline (BMIP): To a two-neck round-bottom flask, 1,10-phenanthroline-5,6-dione (1.68 g, 8 mmol), 2TEG-Bd (4.128 g, 9.6 mmol), ammonium acetate (2.46 g, 32 mmol), and acetic acid (100 mL) were added. The mixture was refluxed under nitrogen atmosphere for 6 h. After the mixture was cooled to room temperature, the solvent was removed by rotary evaporation and the solid was dissolved in water. The solution was extracted with dichloromethane for three times (80 mL × 3), and dichloromethane was removed by rotary evaporation successively. The crude product was purified by column chromatography which used ethyl acetate first, ethyl acetate/methanol (10:1 v/v) successively, and finally methanol as the eluents. The pure product was a light-red gelatinous solid. Yield: 83%. ^1^H NMR (400 MHz, DMSO-*d*_6_, TMS, δ): 3.23 (d, 6H), 3.41–3.45 (m, 4H), 3.52–3.59 (m, 8H), 3.64–3.68 (m, 4H), 3.80–3.86 (td, 4H), 4.21–4.29 (td, 4H), 7.25 (d, 1H), 7.83–7.90 (m, 4H), 8.93 (d, 2H), 9.04 (dd, 2H), 13.59 (s, 1H). ^13^C NMR (400 MHz, DMSO-*d*_6_, δ): 0.57, 39.42, 39.63, 39.84, 40.04, 40.25, 40.46, 40.67, 58.50, 58.53, 68.84, 69.10, 69.46, 69.54, 70.10, 70.36, 70.53, 71.77, 112.81, 114.60, 120.23, 123.58, 130.08, 143.85, 147.87, 148.93. HRMS: 621.29077 [M+H]^+^ (calcd: 620.28).

### Ion Selectivities

The aqueous solution of BMIP (2 mmol/L) was prepared in a volumetric flask (250 mL). Then, to a BMIP solution (1.5 mL), one of different salts (CoCl_2_, CrCl_3_, CuCl_2_, MnCl_2_, NiCl_2_, KCl, LiCl, Na_2_SO_4_, Al (NO_3_)_3_, Pb (NO_3_)_2_, CH_3_COOH, NaH_2_PO_4_, NaHCO_3_, NaHSO_4_, NaNO_2_, NaNO_3_, NaClO_4_, NaBr, NH_4_F, KI, CH_3_COONH_4_, and NaOH) (the final concentration of salts was 3 mol/L) was added and the solution volume was adjusted to be 3 mL, respectively. Finally, the absorption and fluorescence spectra of these mixtures were studied.

### Anti-Interference Experiment

The aqueous solution of BMIP (2 mmol/L) was prepared in a volumetric flask (250 mL). Then, to a BMIP solution (1.5 mL), different salts (NaOH, KCl, Na_2_SO_4_, NaNO_2_, NaNO_3_, NaClO_4_, NaBr, and KI) (the final concentration of each salt was 3 mol/L) were added and the solution volume was adjusted to be 3 mL. This mixture was named S1. To another BMIP solution (1.5 mL), NaOH was added (the final concentration of NaOH was 3 mol/L) and the solution volume was adjusted to be 3 mL. The mixture was named S2. Finally, the fluorescence spectra of these two mixtures were studied.

### Response to Different pH

Aqueous solutions with different pH (neutral water, 10 mol/L H^+^, 6 mol/L H^+^, 2 mol/L H^+^, 1.60, 2.39, 3.31, 4.29, 5.82, 6.36, 8.53, 9.23, 9.89, 11.06, 12.26, 13.11, 13.90, 3 mol/L OHˉ) were prepared in volumetric flasks (10 mL), respectively. Then, BMIP (6.2 mg) was added to these volumetric flasks, respectively. After BMIP dissolved in these solutions, the fluorescence spectra of these mixtures were studied.

### Extreme Alkalinity Detections

Aqueous solutions with different concentrations (0, 1.0, 1.5, 2.0, 2.5, 3.0, 3.5, 4.0, 4.5, 5.0, 5.5, 6.0, 6.5, 7.0, 7.5, 8.0, 8.5, 9.0, 9.5, 10, 10.5, 11, 11.5, 12, 12.5, 13, 13.5, 14, 14.5, 15 mol/L) of NaOH were prepared in volumetric flasks (10 mL), respectively. Then, BMIP (6.2 mg) was added to these volumetric flasks, respectively. After BMIP dissolved in these solutions and reacted with OHˉ, the absorption and fluorescence spectra of these mixtures were studied.

### Repeatability

The aqueous solution of BMIP (2 mmol/L) was prepared in a volumetric flask (250 mL). Then, from this stock solution, four solutions (3 mL) were prepared: (a) BMIP solution (1 mmol/L), (b) BMIP (1 mmol/L) + NaOH (3 mol/L) solution, (c) BMIP solution (1 mmol/L) after adding NaOH (3 mol/L) and then the solution pH became neutral by adding sulfuric acid, and (d) BMIP solution (1 mmol/L) after adding NaOH (3 mol/L), then the solution pH became neutral by adding sulfuric acid and finally adding NaOH (3 mol/L) again. After these four solutions were prepared, their fluorescence spectra were studied.

### AIE Property of BMIPˉ

First, a solution (2 mL) with BMIP (1 mmol/L) and NaOH (3 mol/L) was prepared. Then, NaOH solution (0.1 mL each time, 3 mol/L) was gradually added to the solution. With the increase of NaOH solution, some yellow precipitate dissolved and the other precipitate dispersed. During this process, the fluorescence changes of this solution were studied.

### Reproducibility of the Test Results

To verify the reproducibility of our test results, every experiment was repeated three times. The standard deviations of these tests were calculated and listed in Additional file 1: Table S1. The test results of experiments were almost the same and the standard deviations were low. This indicated that the test results in this work showed good reproducibility.

## Supplementary information

**Figure S1.** The PL spectra of aqueous solutions of BMIP with different concentrations. **Figure S2.** The absorption spectra of aqueous solutions of BMIP with different concentrations. **Figure S3.** The absorption spectra of aqueous solutions of BMIP with different concentrations. **Figure S4.** Photo comparison of aqueous solutions of BMIP before and after the additions of different salts under natural light. **Figure S5.** The absorption spectra of aqueous solutions of BMIP before and after the additions of different salts. **Figure S6.** The PL spectra of aqueous solutions of BMIP after the additions of different salts. **Figure S7.** Photo comparison of aqueous solutions of BMIP with different pH under natural light. **Figure S8.** The absorption spectra of aqueous solutions of BMIP with different pH. **Figure S9.** The PL spectra of aqueous solutions of BMIP with different pH. **Figure S10.** Photo comparison of aqueous solutions of BMIP with different concentrations of OHˉ under natural light. **Figure S11.** Photo comparison of aqueous solutions of BMIP with different concentrations of OHˉ under UV light. **Figure S12.** The PL spectra of aqueous solutions of BMIP with different concentrations of OHˉ. **Figure S13.** The changes of fluorescence intensities of BMIP solutions with different concentrations of OHˉ in water. **Figure S14.** The absorption spectra of aqueous solutions of BMIP with different concentrations of OHˉ. **Table S1.** The standard deviations of every test in this work. **Figure S15.** The ^1^H NMR spectrum of TEG-OTs. **Figure S16.** The ^13^C NMR spectrum of TEG-OTs. **Figure S17.** The mass spectrum of TEG-OTs. **Figure S18.** The ^1^H NMR spectrum of 2TEG-Bd. **Figure S19.** The ^13^C NMR spectrum of 2TEG-Bd. F**igure S20.** The mass spectrum of 2TEG-Bd. **Figure S21.** The ^1^H NMR spectrum of BMIP. **Figure S22.** The ^13^C NMR spectrum of BMIP. **Figure S23.** The mass spectrum of BMIP. (DOCX 13682 kb)

## Data Availability

All data generated or analyzed during this study are included in this published article and its supplementary information files.

## Abbreviations

DMSO: Dimethyl sulfoxide
IP: 1H-imidazo[4,5-f][1,10]phenanthroline
