# Supplementary material for: A Phenanthroline-Based Fluorescent Probe for Highly Selective Detection of Extreme Alkalinity (pH > 14) in Aqueous Solution
Source: Nanoscale Res Lett. 2019 Sep 18;14:318. doi: 10.1186/s11671-019-3149-x (PMC6751243; doi:10.1186/s11671-019-3149-x)
Supplement: Supplementary file 1 — Figure S1. The PL spectra of aqueous solutions of BMIP with different concentrations. Figure S2. The absorption spectra of aqueous solutions of BMIP with different concentrations. Figure S3. The absorption spectra of aqueous solutions of BMIP with different concentrations. Figure S4. Photo comparison of aqueous solutions of BMIP before and after the additions of different salts under natural light. Figure S5. The absorption spectra of aqueous solutions of BMIP before and after the additions of different salts. Figure S6. The PL spectra of aqueous solutions of BMIP after the additions of different salts. Figure S7. Photo comparison of aqueous solutions of BMIP with different pH under natural light. Figure S8. The absorption spectra of aqueous solutions of BMIP with different pH. Figure S9. The PL spectra of aqueous solutions of BMIP with different pH. Figure S10. Photo comparison of aqueous solutions of BMIP with different concentrations of OHˉ under natural light. Figure S11. Photo comparison of aqueous solutions of BMIP with different concentrations of OHˉ under UV light. Figure S12. The PL spectra of aqueous solutions of BMIP with different concentrations of OHˉ. Figure S13. The changes of fluorescence intensities of BMIP solutions with different concentrations of OHˉ in water. Figure S14. The absorption spectra of aqueous solutions of BMIP with different concentrations of OHˉ. Table S1. The standard deviations of every test in this work. Figure S15. The 1H NMR spectrum of TEG-OTs. Figure S16. The 13C NMR spectrum of TEG-OTs. Figure S17. The mass spectrum of TEG-OTs. Figure S18. The 1H NMR spectrum of 2TEG-Bd. Figure S19. The 13C NMR spectrum of 2TEG-Bd. Figure S20. The mass spectrum of 2TEG-Bd. Figure S21. The 1H NMR spectrum of BMIP. Figure S22. The 13C NMR spectrum of BMIP. Figure S23. The mass spectrum of BMIP. (DOCX 13682 kb) [file 11671_2019_3149_MOESM1_ESM.docx]

**Supporting Information**

**A Phenanthroline-Based Fluorescent Probe for Highly Selective Detection of Extreme Alkalinity (pH > 14) in Aqueous Solution**

**Xiaoyu Ma^1,2^, Shanyong Chen^1*^, Hong Yu^1*^, Youwei Guan^1^, Junjun Li^1^, Xingwu Yan^1^, Zhenghao Zhang^3^**

^1^Research Institute for New Materials Technology, Chongqing University of Arts and Sciences, Yongchuan 402160, P. R. China. E-mail: jluchensy@163.com (S. Y. Chen) and hongyu@cqwu.edu.cn (H. Yu)

^2^College of Chemistry, Jilin University, Changchun 130012, P. R. China.

^3^State Grid Tianjin Electric Power Corporation Chengxi District Supply Company, Tianjin 300191, P. R. China

**Experimental data**


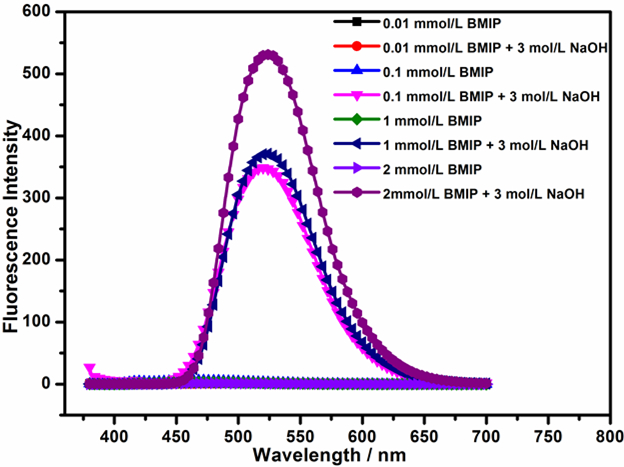


**Fig. S1** The PL spectra of aqueous solutions (concentration of OHˉ: 3 mol/L) of BMIP with different concentrations (0.01, 0.1, 1 and 2 mmol/L respectively).





**Fig. S2** The absorption spectra of aqueous solutions (concentration of OHˉ: 3 mol/L) of BMIP with different concentrations (0.01, 0.1, 1 and 2 mmol/L respectively).





**Fig. S3** The absorption spectra of aqueous solutions (concentration of OHˉ: 3 mol/L) of BMIP with different concentrations (0.01 and 0.1 mmol/L respectively).


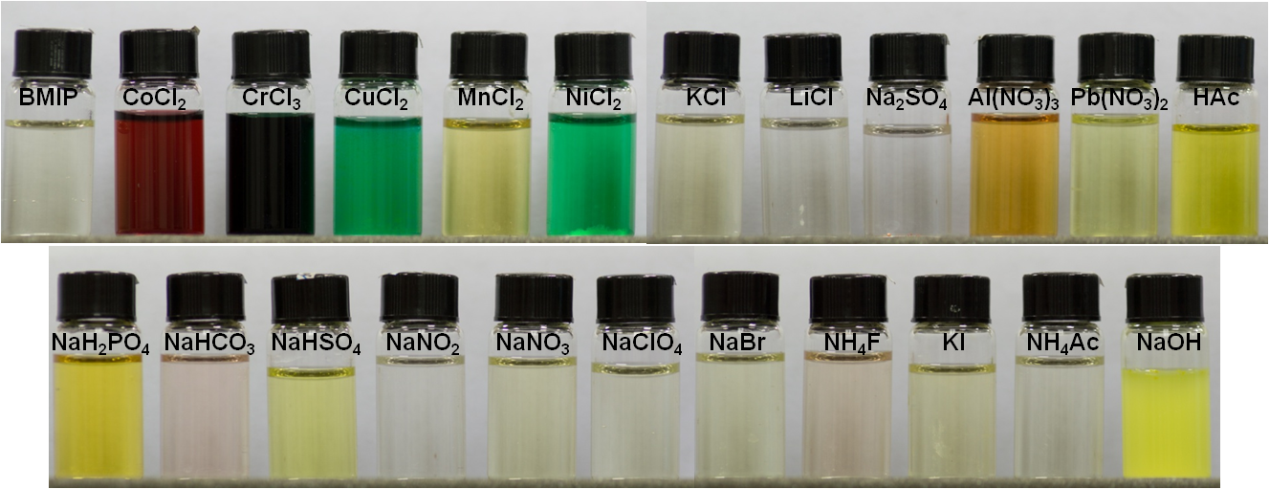


**Fig. S4** Photo comparison of aqueous solutions (1 mmol/L) of BMIP before and after the additions of different salts (CoCl_2_, CrCl_3_, CuCl_2_, MnCl_2_, NiCl_2_, KCl, LiCl, Na_2_SO_4_, Al(NO_3_)_3_, Pb(NO_3_)_2_, CH_3_COOH, NaH_2_PO_4_, NaHCO_3_, NaHSO_4_, NaNO_2_, NaNO_3_, NaClO_4_, NaBr, NH_4_F, KI, CH_3_COONH_4_, NaOH respectively) (3 mol/L) under natural light.









**Fig. S5** (a) The absorption spectra of aqueous solutions (1 mmol/L) of BMIP before and after the additions of different salts (CoCl_2_, CrCl_3_, CuCl_2_, MnCl_2_, NiCl_2_, KCl, LiCl, Na_2_SO_4_, Al(NO_3_)_3_, Pb(NO_3_)_2_, CH_3_COOH, NaH_2_PO_4_, NaHCO_3_, NaHSO_4_, NaNO_2_, NaNO_3_, NaClO_4_, NaBr, NH_4_F, KI, CH_3_COONH_4_, NaOH respectively) (3 mol/L). (b) and (c) The detailed absorption spectra of aqueous solutions (1 mmol/L) of BMIP before and after the additions of different salts.






**Fig. S6** The PL spectra of aqueous solutions (1 mmol/L) of BMIP after the additions of different salts (a) CoCl_2_, CrCl_3_, CuCl_2_, MnCl_2_, NiCl_2_, KCl, LiCl, Na_2_SO_4_, Al(NO_3_)_3_, Pb(NO_3_)_2_, CH_3_COOH respectively (3 mol/L), (b) none, NaH_2_PO_4_, NaHCO_3_, NaHSO_4_, NaNO_2_, NaNO_3_, NaClO_4_, NaBr, NH_4_F, KI, CH_3_COONH_4_ respectively (3 mol/L).


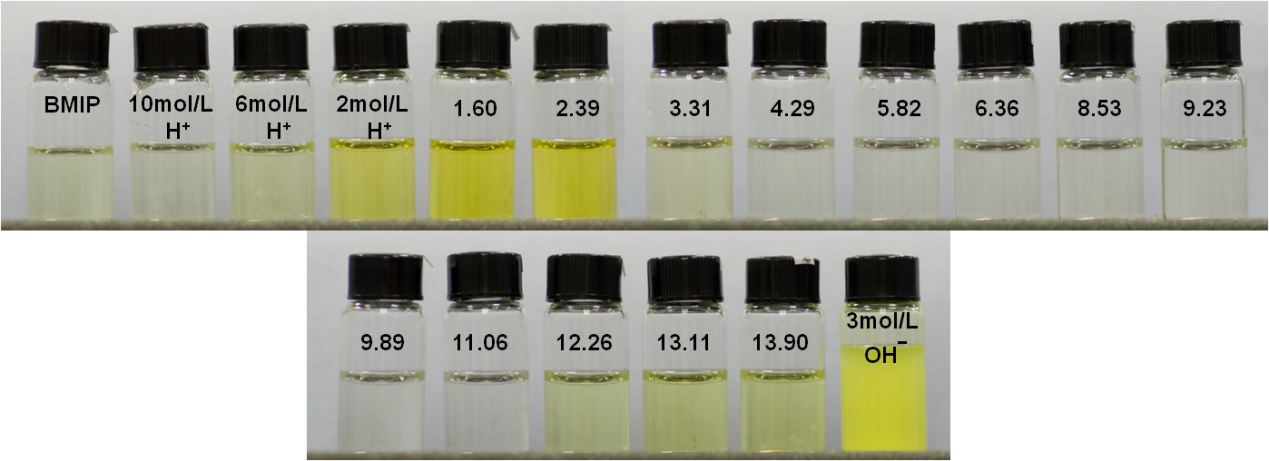


**Fig. S7** Photo comparison of aqueous solutions (1 mmol/L) of BMIP with different pH (neutral water, 10 mol/L H^+^, 6 mol/L H^+^, 2 mol/L H^+^, 1.60, 2.39, 3.31, 4.29, 5.82, 6.36, 8.53, 9.23, 9.89, 11.06, 12.26, 13.11, 13.90, 3 mol/L OHˉ respectively) under natural light.










**Fig. S8** (a) The absorption spectra of aqueous solutions (1 mmol/L) of BMIP with different pH (neutral water, 10 mol/L H^+^, 6 mol/L H^+^, 2 mol/L H^+^, 1.60, 2.39, 3.31, 4.29, 5.82, 6.36, 8.53, 9.23, 9.89, 11.06, 12.26, 13.11, 13.90, 3 mol/L OHˉ respectively). (b) and (c) The detailed absorption spectra of aqueous solutions (1 mmol/L) of BMIP with different pH.






**Fig. S9** (a) The PL spectra of aqueous solutions (1 mmol/L) of BMIP with different pH (neutral water, 10 mol/L H^+^, 6 mol/L H^+^, 2 mol/L H^+^, 1.60, 2.39, 3.31, 4.29, 5.82 respectively). (b) The PL spectra of aqueous solutions (1 mmol/L) of BMIP with different pH (6.36, 8.53, 9.23, 9.89, 11.06, 12.26, 13.11, 13.90, 3 mol/L OHˉ respectively).


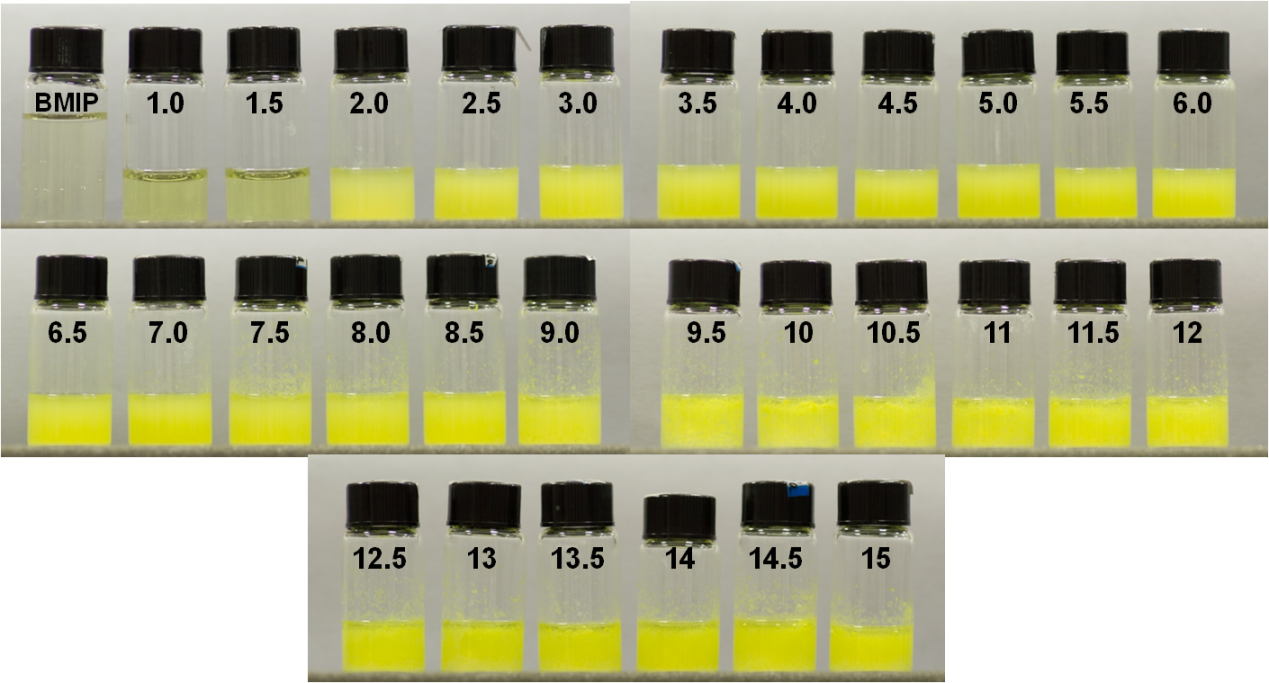


**Fig. S10** Photo comparison of aqueous solutions (1 mmol/L) of BMIP with different concentrations (0, 1.0, 1.5, 2.0, 2.5, 3.0, 3.5, 4.0, 4.5, 5.0, 5.5, 6.0, 6.5, 7.0, 7.5, 8.0, 8.5, 9.0, 9.5, 10, 10.5, 11, 11.5, 12, 12.5, 13, 13.5, 14, 14.5, 15 mol/L respectively) of OHˉ under natural light.


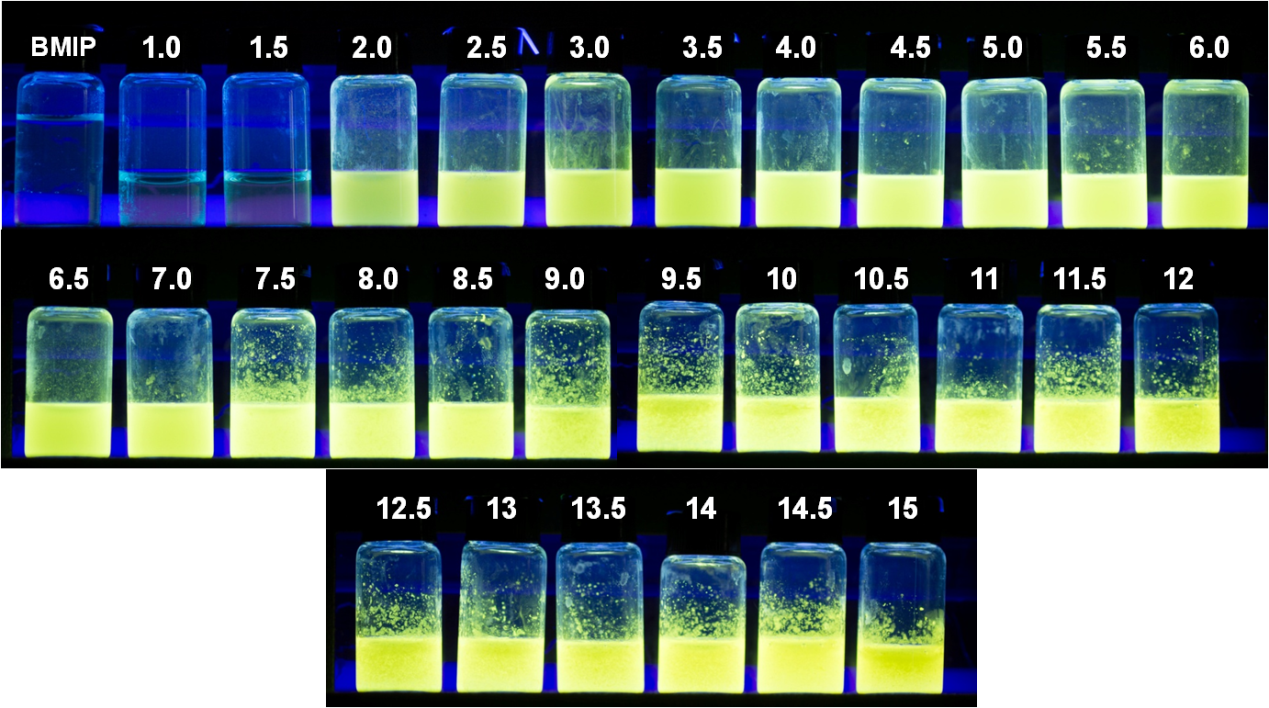


**Fig. S11** Photo comparison of aqueous solutions (1 mmol/L) of BMIP with different concentrations (0, 1.0, 1.5, 2.0, 2.5, 3.0, 3.5, 4.0, 4.5, 5.0, 5.5, 6.0, 6.5, 7.0, 7.5, 8.0, 8.5, 9.0, 9.5, 10, 10.5, 11, 11.5, 12, 12.5, 13, 13.5, 14, 14.5, 15 mol/L respectively) of OHˉ under UV light (365 nm).











**Fig. S12** (a) The PL spectra of aqueous solutions (1 mmol/L) of BMIP with different concentrations (0, 1.0, 1.5, 2.0, 2.5, 3.0, 3.5, 4.0, 4.5, 5.0, 5.5, 6.0, 6.5, 7.0, 7.5, 8.0, 8.5, 9.0, 9.5, 10, 10.5, 11, 11.5, 12, 12.5, 13, 13.5, 14, 14.5, 15 mol/L respectively) of OHˉ. (b) and (c) The PL spectra of aqueous solutions (1 mmol/L) of BMIP with different concentrations of OHˉ.





**Fig. S13** The changes of fluorescence intensities of BMIP solutions (1 mmol/L) with different concentrations (0, 1.0, 1.5, 2.0, 2.5, 3.0, 3.5, 4.0, 4.5, 5.0, 5.5, 6.0, 6.5, 7.0, 7.5, 8.0, 8.5, 9.0, 9.5, 10, 10.5, 11, 11.5, 12, 12.5, 13, 13.5, 14, 14.5, 15 mol/L respectively) of OHˉ in water.














**Fig. S14** (a) The absorption spectra of aqueous solutions (1 mmol/L) of BMIP with different concentrations (0, 1.0, 1.5, 2.0, 2.5, 3.0, 3.5, 4.0, 4.5, 5.0, 5.5, 6.0, 6.5, 7.0, 7.5, 8.0, 8.5, 9.0, 9.5, 10, 10.5, 11, 11.5, 12, 12.5, 13, 13.5, 14, 14.5, 15 mol/L respectively) of OHˉ. (b), (c) and (d) The PL spectra of aqueous solutions (1 mmol/L) of BMIP with different concentrations of OHˉ.

**Table S1** The standard deviations (SD) of every test in this work.

| ions | salts | none | CoCl_2_ | CrCl_3_ | CuCl_2_ | MnCl_2_ | NiCl_2_ | KCl | LiCl | Na_2_SO_4_ |
| --- | --- | --- | --- | --- | --- | --- | --- | --- | --- | --- |
|  | SD | 0.0085 | 0.000084 | 0 | 0.000076 | 0.000065 | 0.00060 | 0.019 | 0.045 | 0.12 |
|  | salts | Al(NO_3_)_3_ | Pb(NO_3_)_2_ | CH_3_COOH | NaH_2_PO_4_ | NaHCO_3_ | NaHSO_4_ | NaNO_2_ | NaNO_3_ | NaClO_4_ |
|  | SD | 0.0082 | 0.012 | 0.011 | 0.0060 | 0.060 | 0.0065 | 0.000080 | 0.021 | 0.035 |
|  | salts | NaBr | NH_4_F | KI | CH_3_COONH_4_ | NaOH |  |  |  |  |
|  | SD | 0.042 | 0.0058 | 0.012 | 0.0081 | 0.40 |  |  |  |  |
| Anti-  interf  erence |  | BMIP | BMIP+NaOH | | BMIP+NaOH+KCl+Na_2_SO_4_+NaNO_2_+NaNO_3_+NaClO_4_+NaBr+KI | | | | | |
|  | SD | 0.0085 | 0.4041 | | 1.37 | | | | | |
| pH | pH | neutral water | 10 mol/L H^+^ | 6 mol/L H^+^ | 2 mol/L H^+^ | 1.60 | 2.39 | 3.31 | 4.29 | 5.82 |
|  | SD | 0.0085 | 0.0049 | 0.0035 | 0.0090 | 0.0096 | 0.0081 | 0.02 | 0.042 | 0.051 |
|  | pH | 6.36 | 8.53 | 9.23 | 9.89 | 11.06 | 12.26 | 13.11 | 13.90 | 3 mol/L  OHˉ |
|  | SD | 0.040 | 0.042 | 0.045 | 0.051 | 0.0058 | 0.0072 | 0.0065 | 0.0070 | 0.40 |
| OHˉ | OHˉ | 0 | 1.0 | 1.5 | 2.0 | 2.5 | 3.0 | 3.5 | 4.0 | 4.5 |
|  | SD | 0.0085 | 0.0080 | 0.0056 | 1.10 | 1.05 | 0.40 | 0.92 | 1.31 | 1.63 |
|  | OHˉ | 5.0 | 5.5 | 6.0 | 6.5 | 7.0 | 7.5 | 8.0 | 8.5 | 9.0 |
|  | SD | 1.58 | 2.05 | 2.17 | 2.10 | 1.75 | 1.82 | 1.52 | 1.46 | 1.33 |
|  | OHˉ | 9.5 | 10 | 10.5 | 11 | 11.5 | 12 | 12.5 | 13 | 13.5 |
|  | SD | 1.06 | 1.16 | 0.87 | 1.15 | 0.95 | 1.22 | 0.63 | 0.79 | 0.98 |
|  | OHˉ | 14 | 14.5 | 15 |  |  |  |  |  |  |
|  | SD | 0.89 | 1.01 | 1.07 |  |  |  |  |  |  |
| time | time | 10 s | 46 s | 83 s | 116 s | 147 s | 179 s | 211 s | 240 s | 275 s |
|  | SD | 0.75 | 0.60 | 0.81 | 0.52 | 0.46 | 0.68 | 0.36 | 0.91 | 0.86 |
|  | time | 307 s | 337 s | 369 s |  |  |  |  |  |  |
|  | SD | 0.62 | 0.88 | 0.78 |  |  |  |  |  |  |
| AIE | mL | 0 | 0.1 | 0.2 | 0.3 | 0.4 | 0.5 | 0.6 | 0.7 | 0.8 |
|  | SD | 0.40 | 0.86 | 0.66 | 0.56 | 0.55 | 0.52 | 0.52 | 0.39 | 0.37 |
|  | mL | 0.9 |  |  |  |  |  |  |  |  |
|  | SD | 0.35 |  |  |  |  |  |  |  |  |


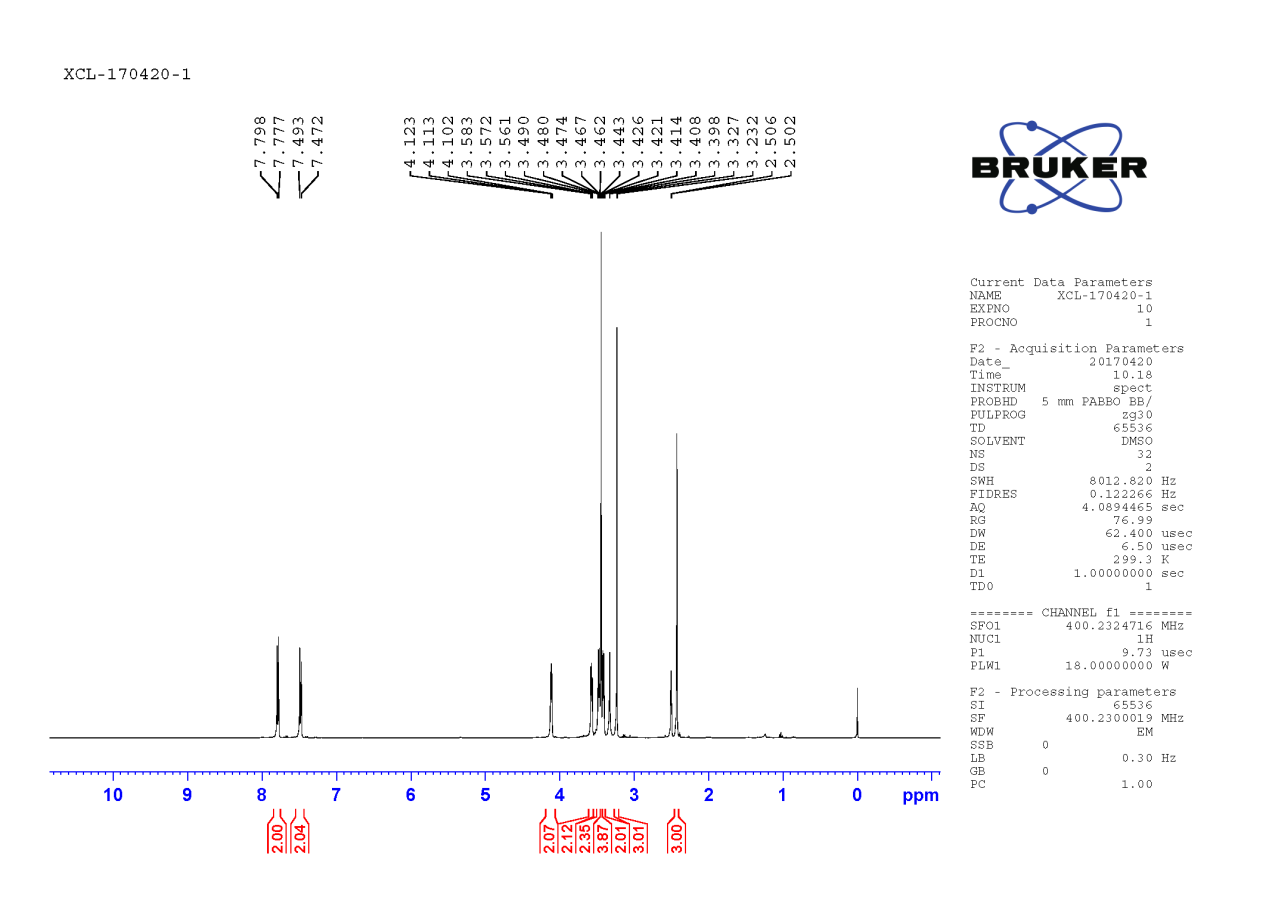


**Fig. S15** The ^1^H NMR spectrum of TEG-OTs in DMSO-*d*_6_.


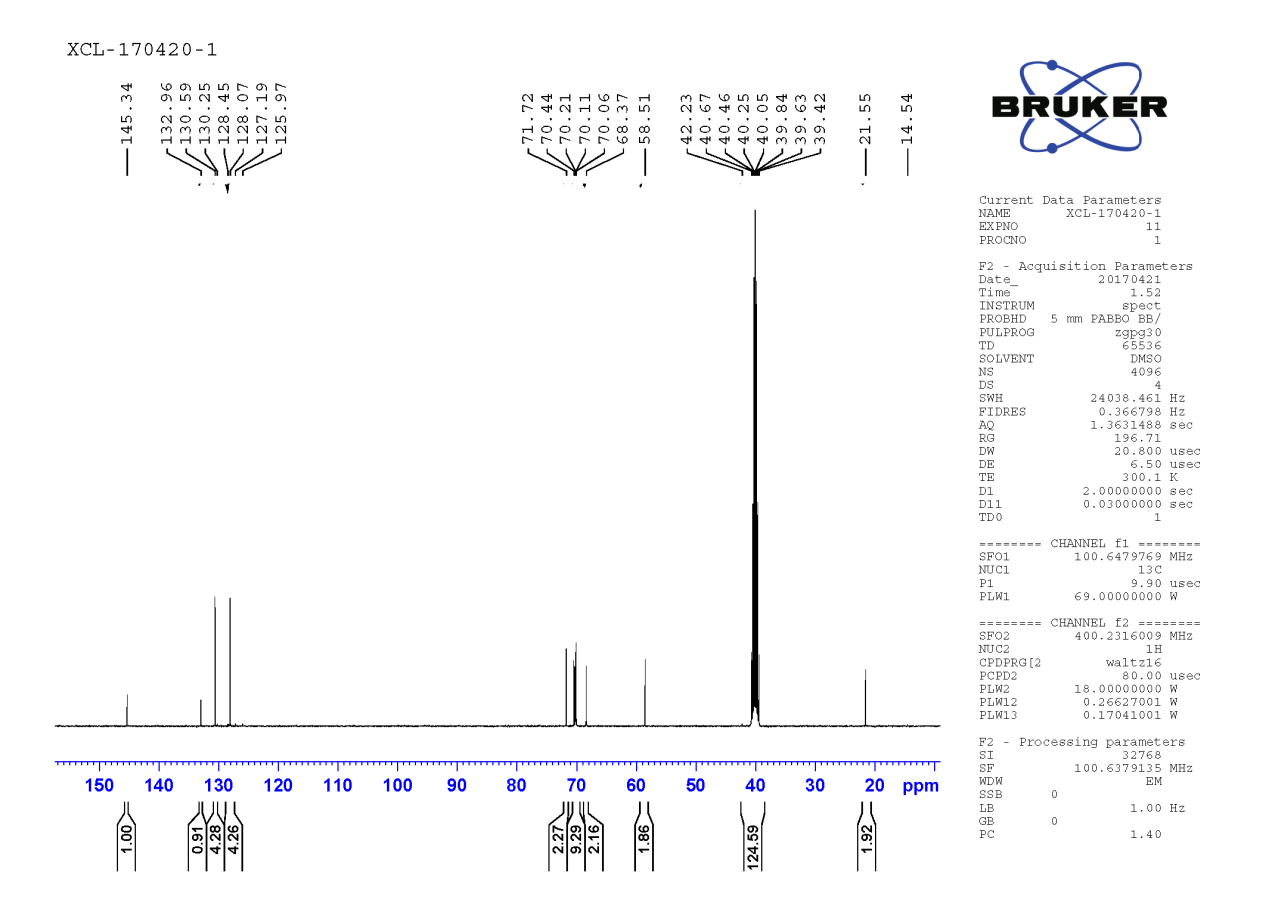


**Fig. S16** The ^13^C NMR spectrum of TEG-OTs in DMSO-*d*_6_.


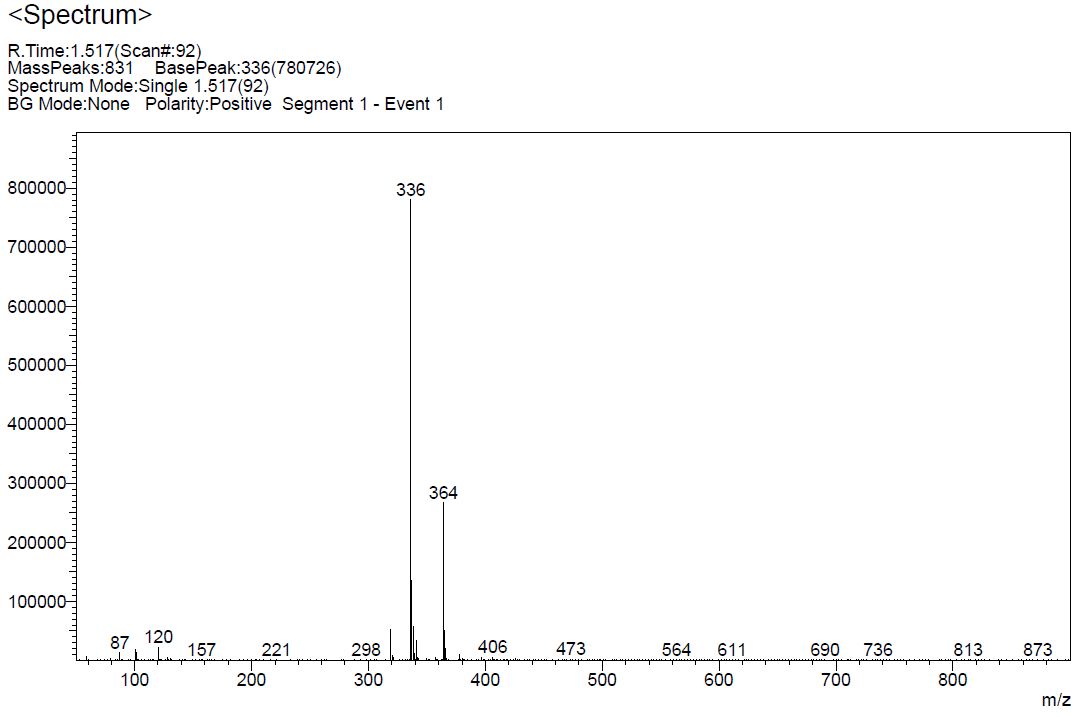


**Fig. S17** The mass spectrum of TEG-OTs.


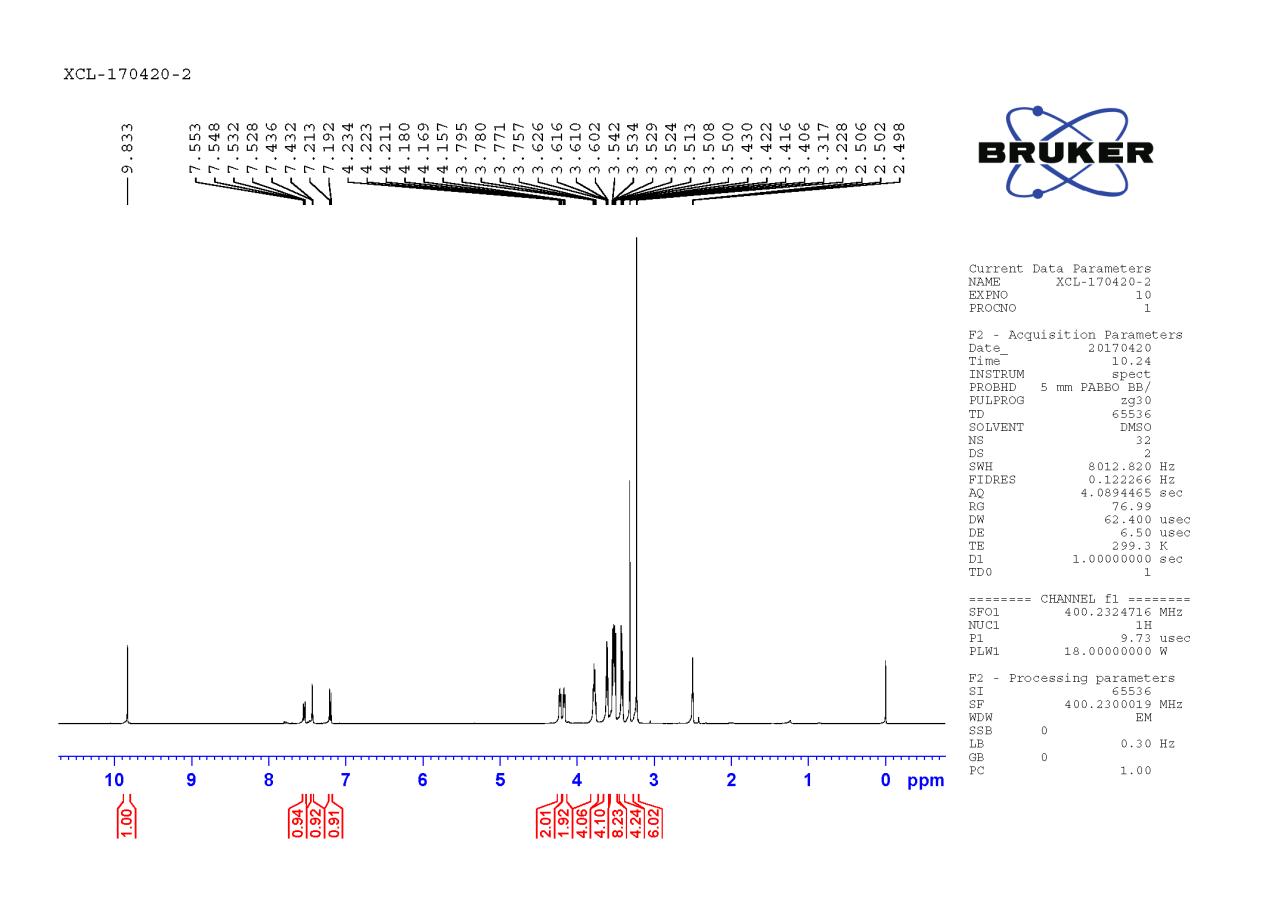


**Fig. S18** The ^1^H NMR spectrum of 2TEG-Bd in DMSO-*d*_6_.


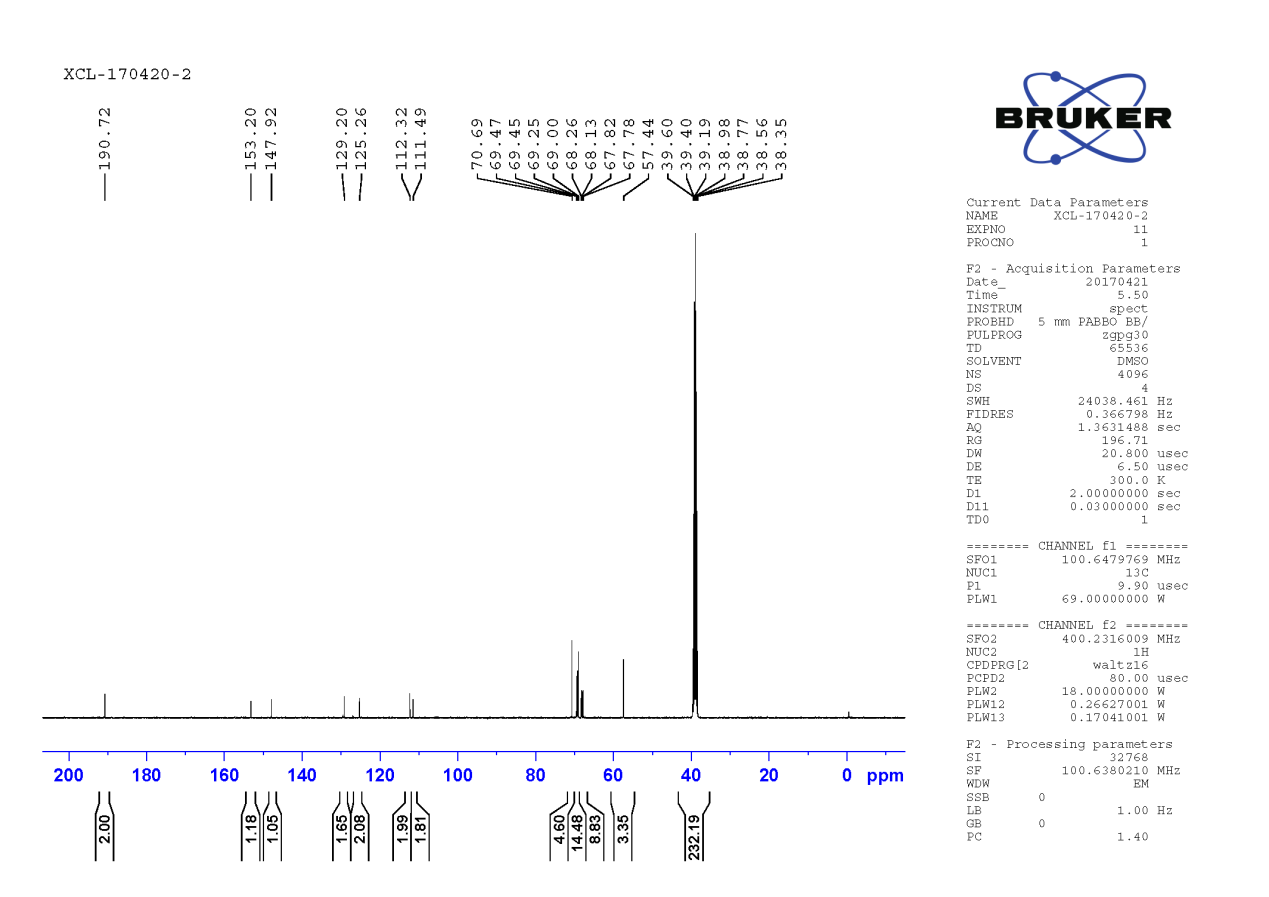


**Fig. S19** The ^13^C NMR spectrum of 2TEG-Bd in DMSO-*d*_6_.


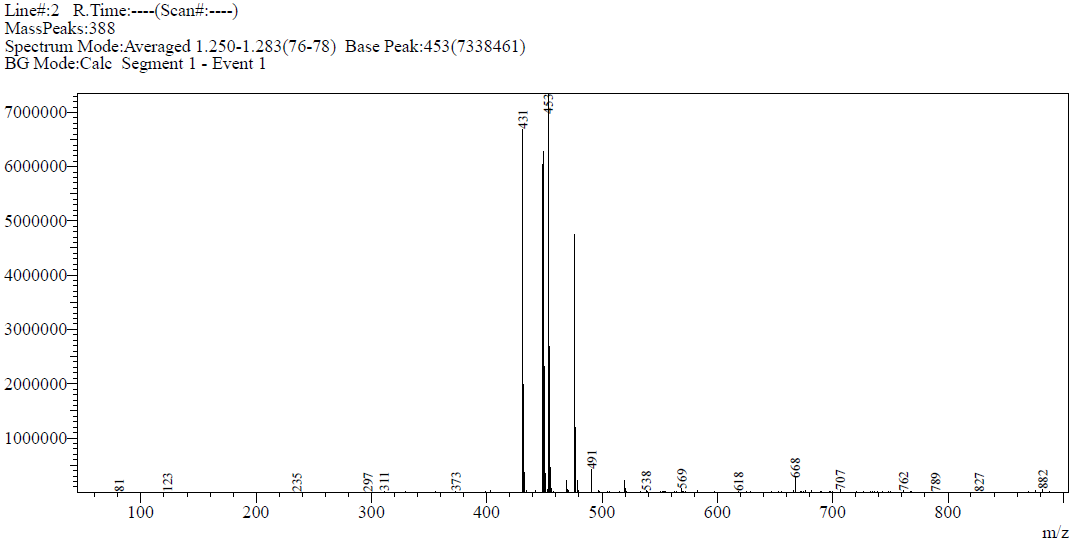


**Fig. S20** The mass spectrum of 2TEG-Bd.


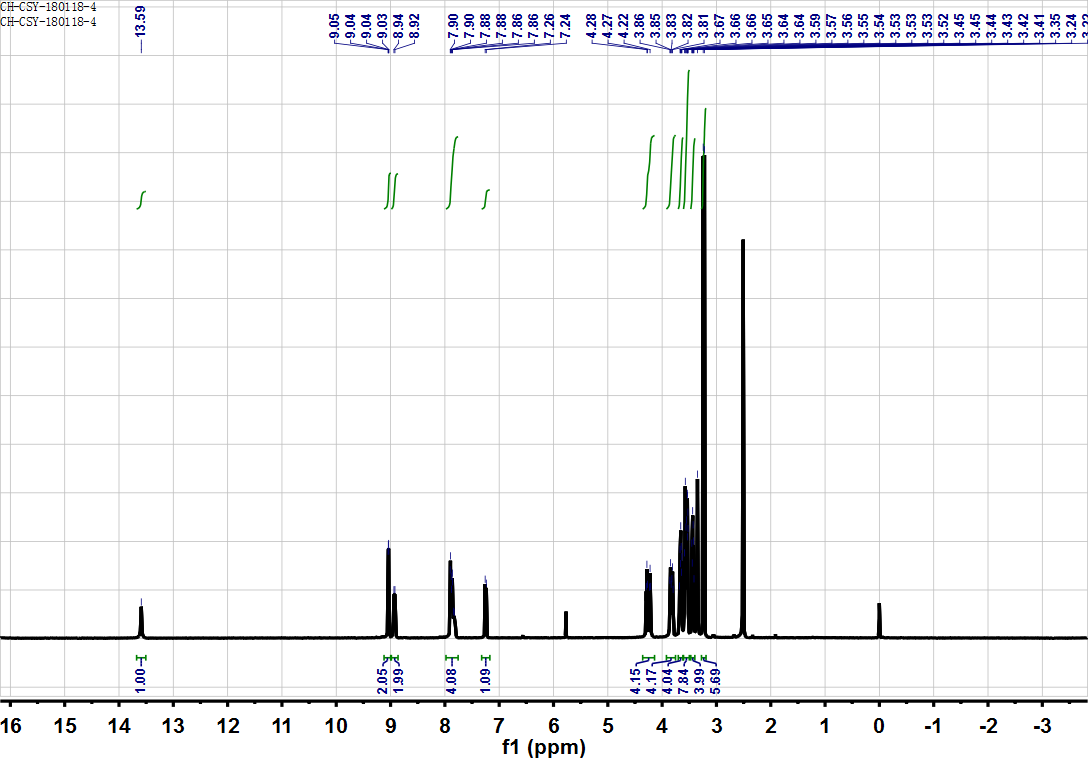


**Fig. S21** The ^1^H NMR spectrum of BMIP in DMSO-*d*_6_.


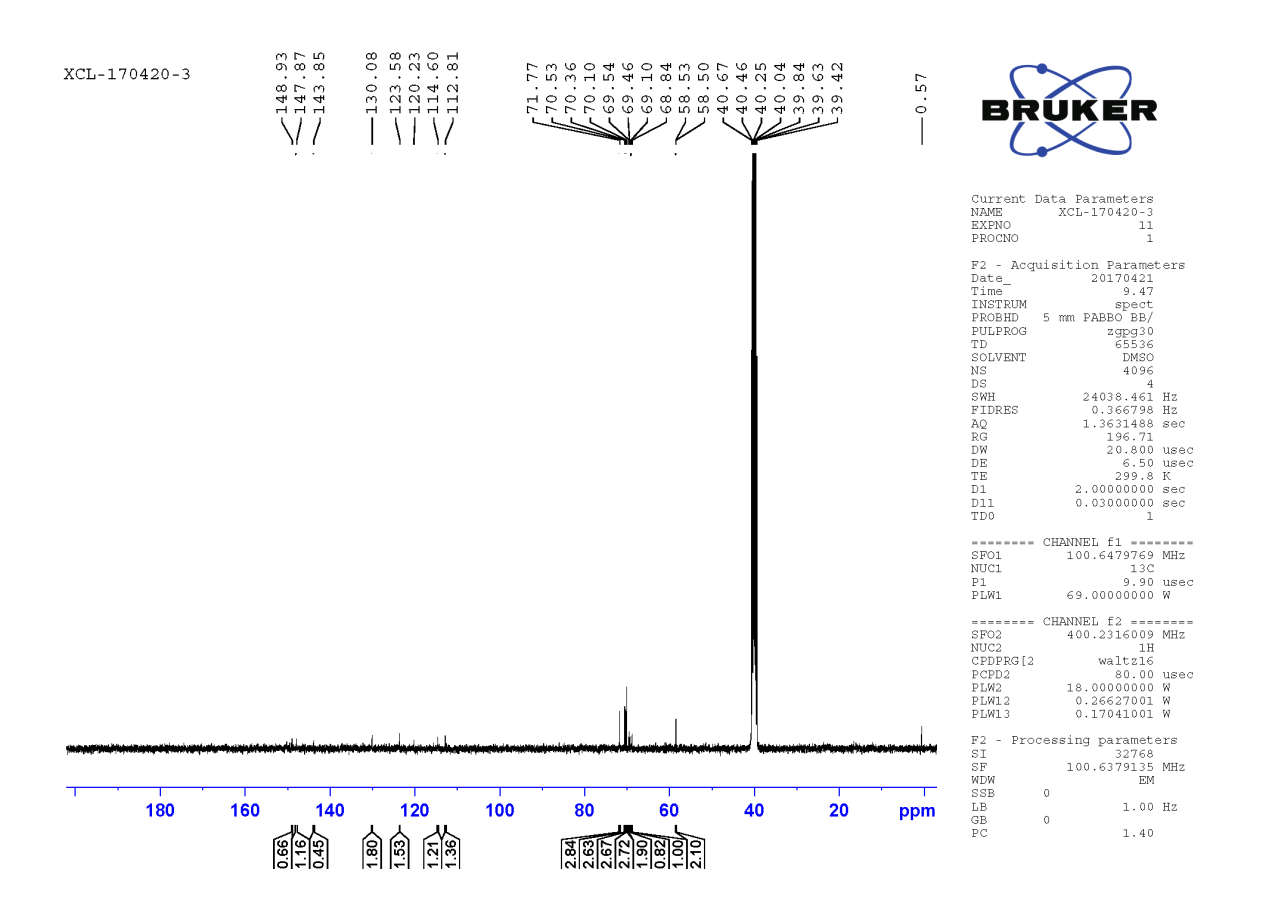


**Fig. S22** The ^13^C NMR spectrum of BMIP in DMSO-*d*_6_.

**Fig. S23** The mass spectrum of BMIP.
